# Supplementary material for: Structural and functional insights into the Rcs phosphorelay
Source: bioRxiv. 2026 May 9:2026.05.08.723598. Preprint. [Version 1] doi: 10.64898/2026.05.08.723598 (PMC13317618; doi:10.64898/2026.05.08.723598)
Supplement: Supplement 1 [file media-1.pdf]

Structural and functional insights into the Rcs phosphorelay

Melesse Nune[1,3]†, Anushya Petchiappan[2]†, Istvan Botos[3], Nadim Majdalani[2], Sydney H. Shapiro[3], Rodolfo Ghirlando[3], Chin-Hsien Tai[2], Amila Abeykoon[3], Ann Marie Stanley[3], Bridgette M. Beach[3], Susan Gottesman[2*], Susan K. Buchanan[3*]

[1] National Institutes of General and Medical Sciences, National Institutes of Health (NIH/NIGMS), Bethesda, Maryland, USA

[2] Center for Cancer Research, National Cancer Institute, Bethesda, Maryland, USA

[3] Laboratory of Molecular Biology, National Institutes of Diabetes and Digestive Diseases, National Institutes of Health (NIH/NIDDK), Bethesda, Maryland, USA

This document includes:
Supplementary tables, figures and figure legends

A

Protein	Size (a.a)	% Identity	% Similarity
RcsB$_{Kp}$/RcsB$_{Ec}$	216/216	97	98
RcsF$_{Kp}$/RcsF$_{Ec}$	135/134	83	93.3
IgaA$_{Kp}$/IgaA$_{Ec}$	709/711	72.2	84.2
RcsD$_{Kp}$/RcsD$_{Ec}$	885/890	71.6	85
RcsC$_{Kp}$/RcsC$_{Ec}$	946/949	79.7	88.1

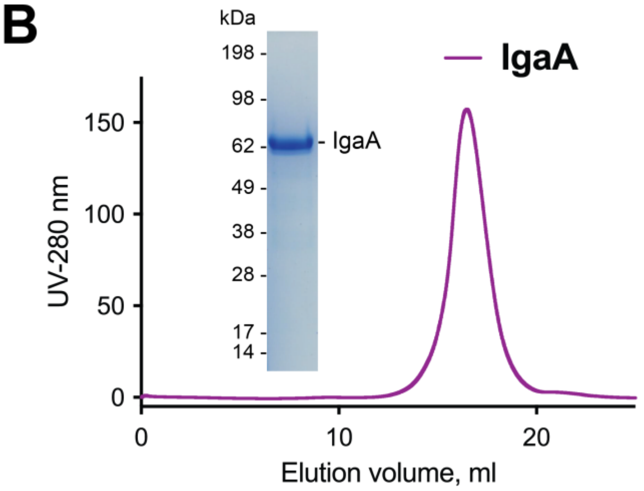

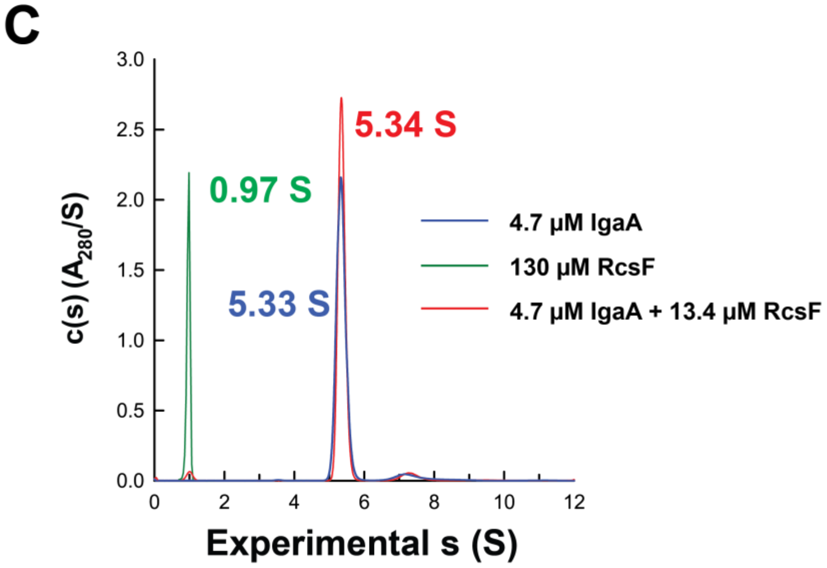

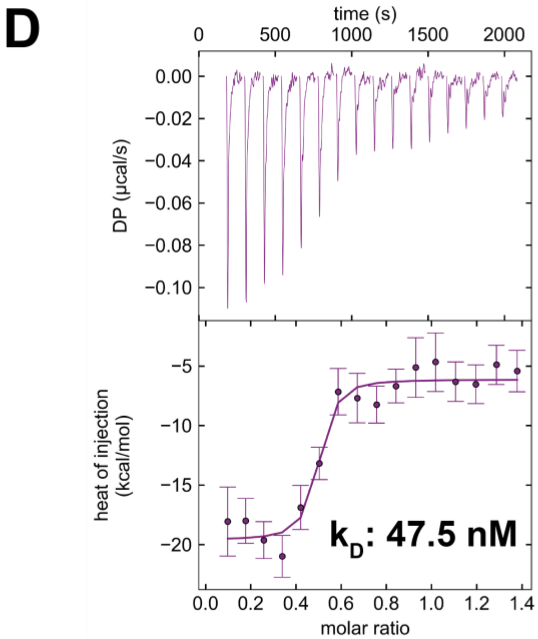

Figure S1: A) Sequence conservation of Rcs proteins. The proteins from *E. coli* and *K. pneumoniae* were analyzed using EMBOSS NEEDLE pairwise sequence alignment tool[1]. **B)** SEC and SDS-PAGE gel analysis of purified full-length IgaA. **C**) AUC results corresponding to panel A, including results for Apo RcsF and IgaA/RcsF complex. **D**) ITC results showing the binding interaction between full-length IgaA$_{Kp}$ and RcsF$_{Kp}$.

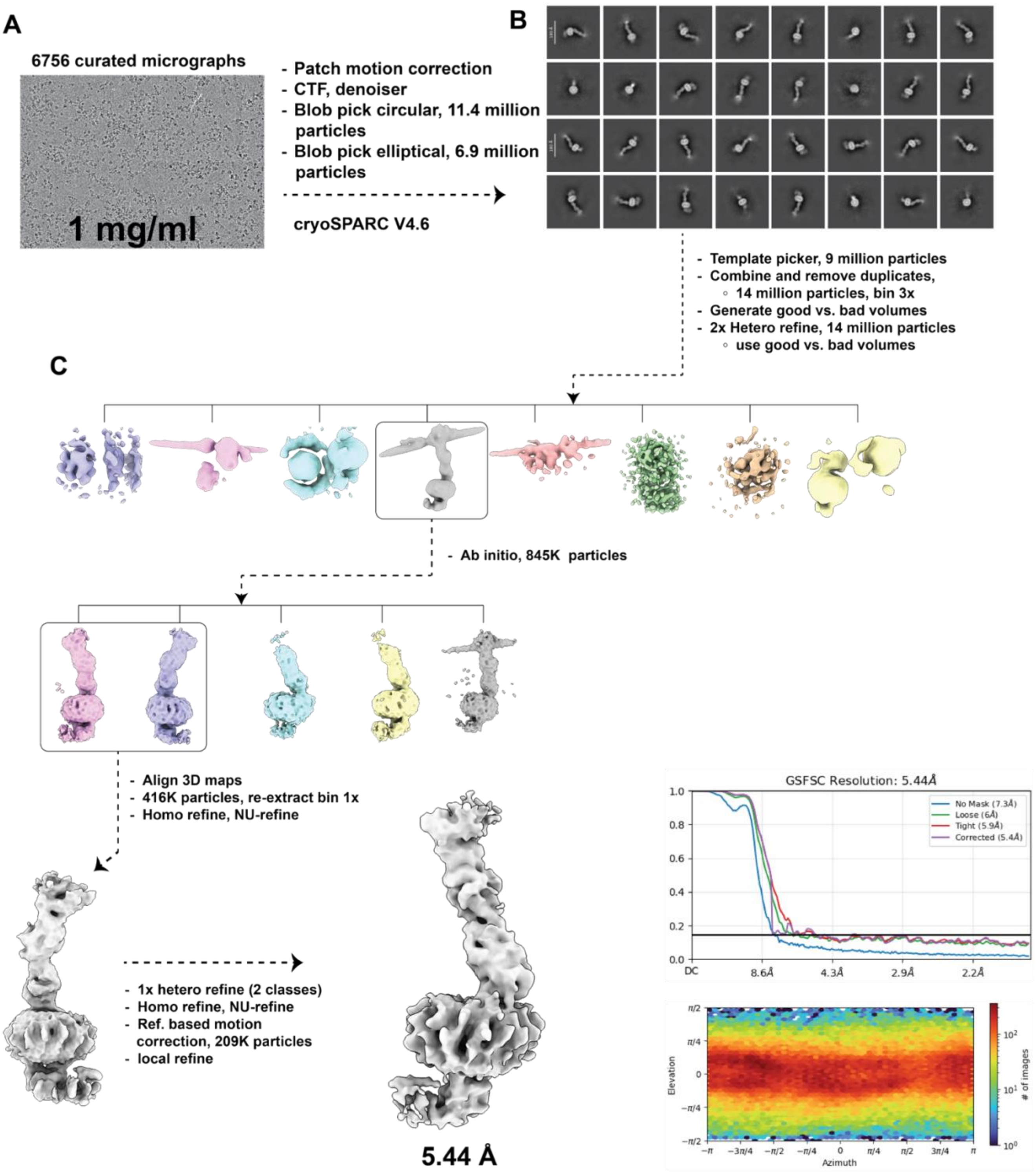

Figure S2: Overview of the IgaA/RcsF data-processing workflow. A) Representative motion-corrected micrograph. B) Selected 2D class averages. C) Schematic representation of the Cryo-EM data-processing pipeline for IgaA/RcsF complex. This includes the Fourier correlation plot (GFSC) showing the map quality and resolution and viewing direction plot (Azimuth) illustrating the orientation diversity of the data used to construct the structure.

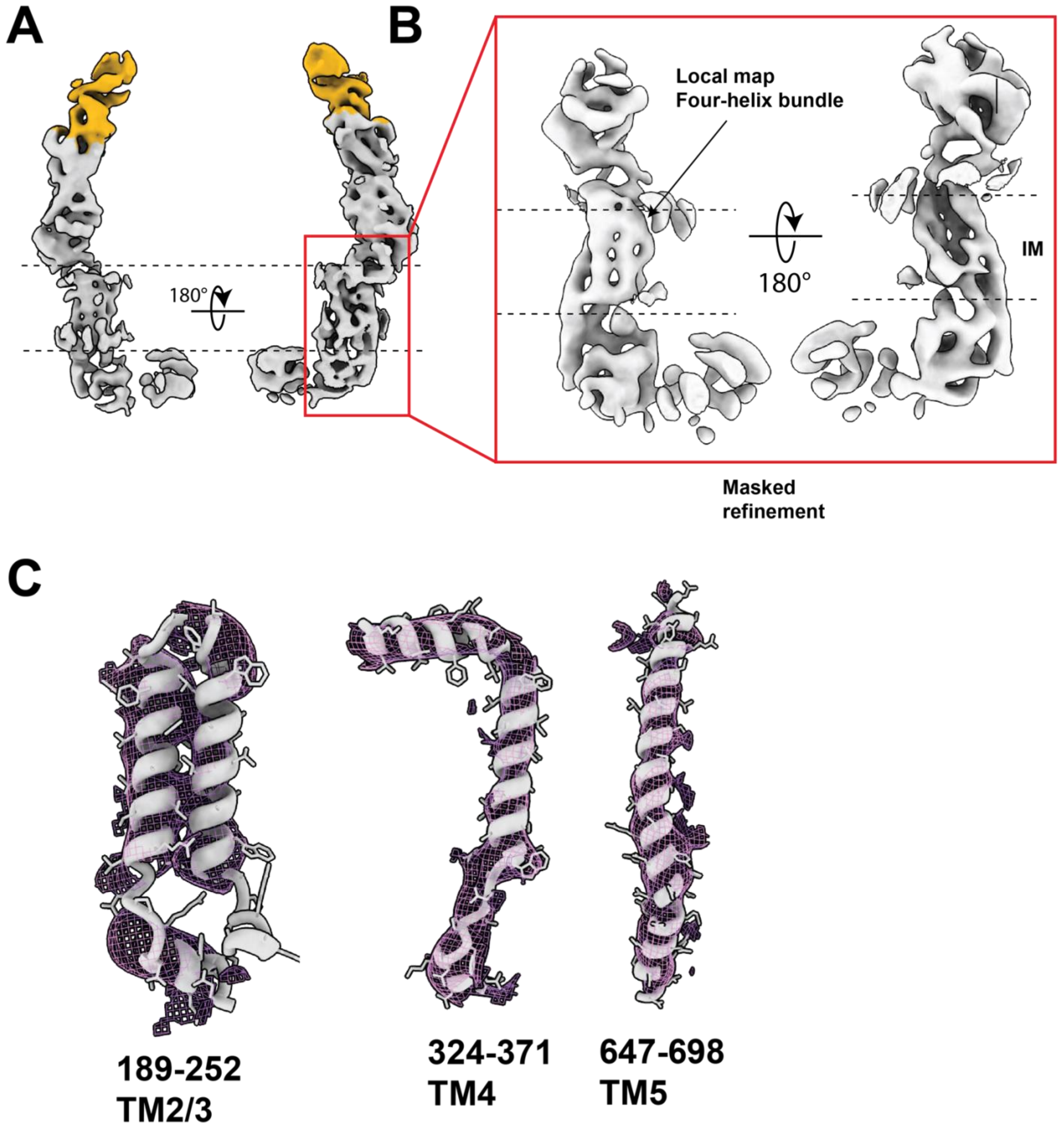

Figure S3: Detailed views of IgaA/RcsF structure. A) Same as in Fig. 2A but with the detergent micelle removed for improved clarity. B) Detailed views of the transmembrane domains obtained through masked refinement. C) Density maps and model fits for IgaA transmembrane domains; four of the five transmembrane domains have been successfully identified.

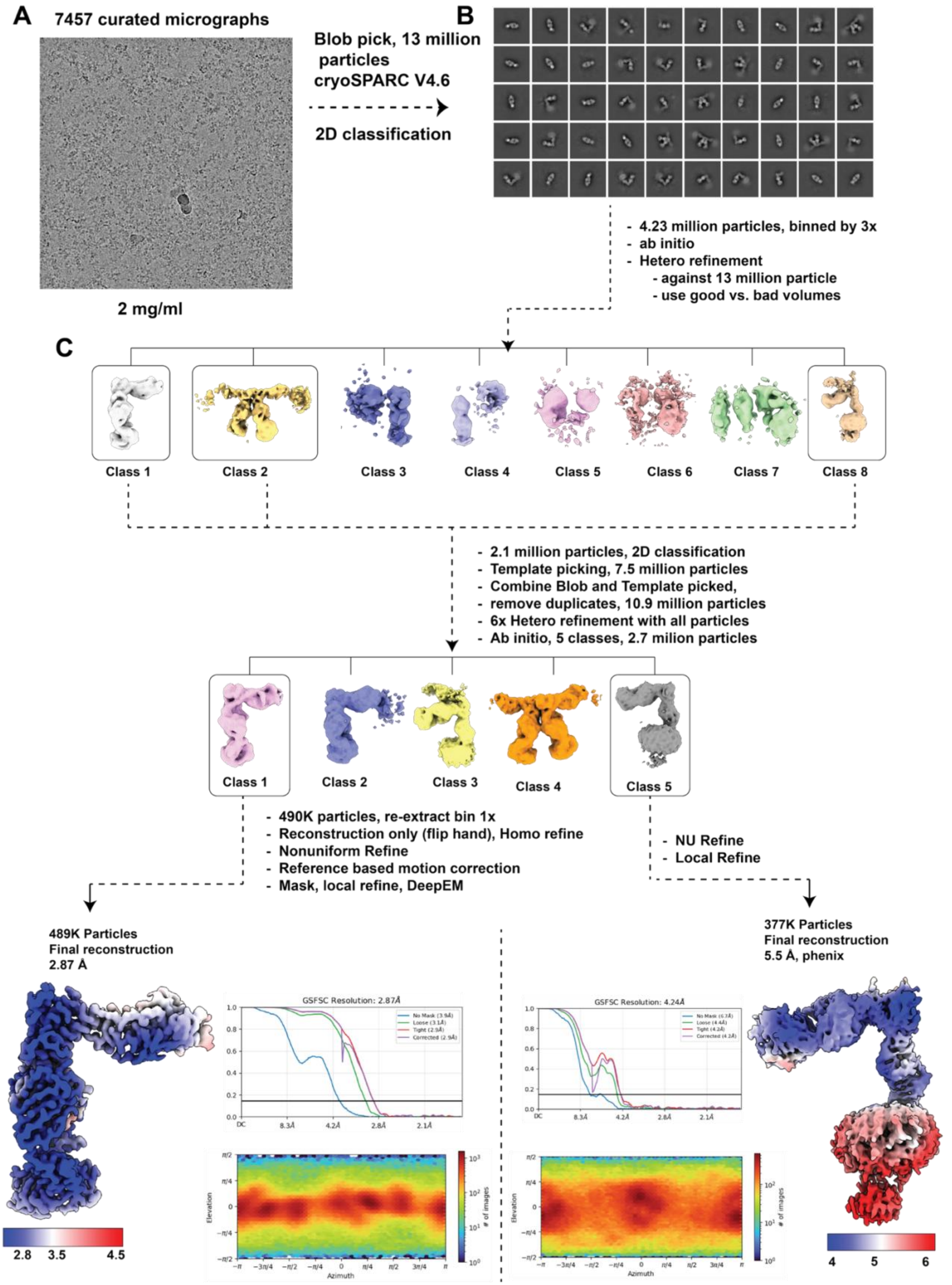

Figure S4: overview of IgaA/Fab57 data processing workflow. A) Representative motion-corrected micrograph. B) Selected 2D class averages used for further analysis. C) Schematic representation of the Cryo-EM data processing pipeline, including FSC curves and azimuth plots. It is important to note that the resolution estimation for the full-length map obtained from cryosparc appears to be overestimated, therefore, we report the unmasked resolution.

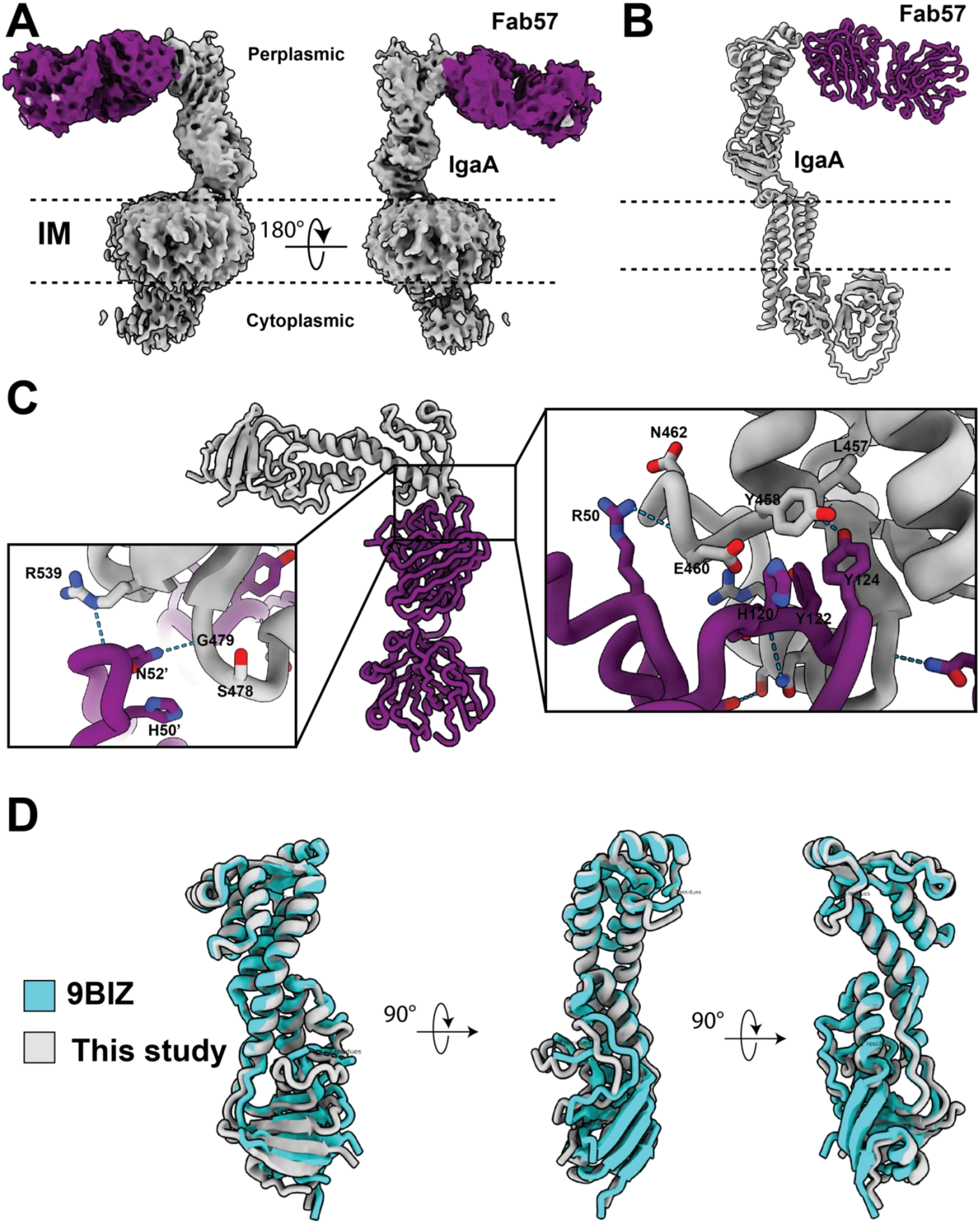

A
Fab57
Perplasmic
IgaA
IM
180°
Cytoplasmic
B
Fab57
IgaA
C
N462
L457
R50
Y458
E460
Y124
H120
Y122
R539
G479
N52'
S478
H50'
D
9BIZ
This study
90°
90°

Figure S5: Detailed views of IgaA/Fab57 binding interface. A) Multiple views of maps of the full-length IgaA in complex with Fab57. B) Alphafold3 model of apo IgaA and Fab57 fitted into the cryoEM map in panel A. C) Atomic-level views of the IgaA/Fab57 binding interface. D) Superposition of the crystal structure of IgaA (9BIZ) with IgaA structure from Fab57 complex; the overall rmsd is 0.89 Å.

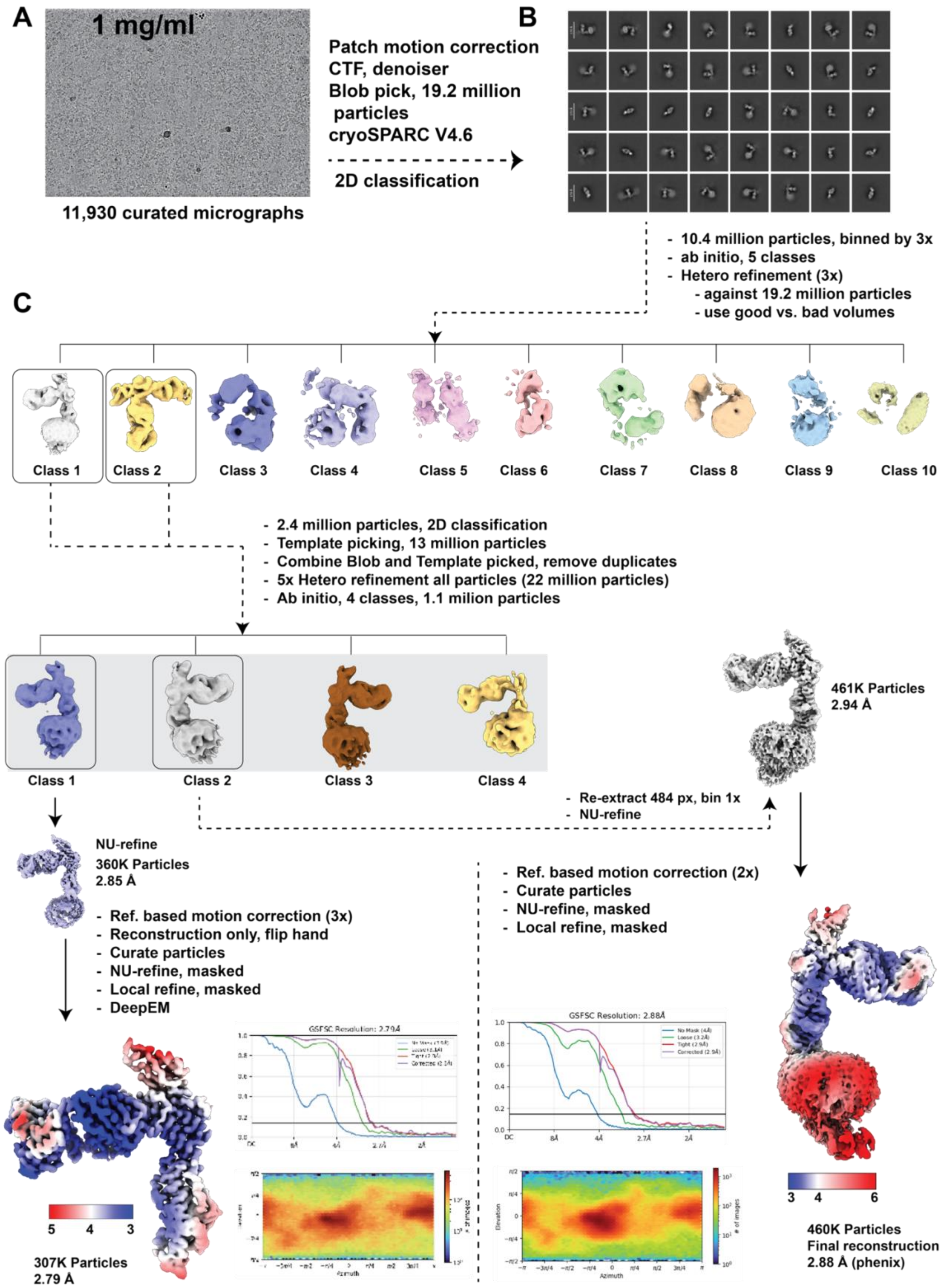

Figure S6: Overview of the IgaA/Fab57/RcsF data-processing workflow. A) Representative motion-corrected micrograph of IgaA/Fab57/RcsF particles. B) Selected 2D class averages that highlight distinct particle features used for further analysis. C) A schematic representation of the Cryo-EM data-processing pipeline, outlining key steps from particle picking to reconstruction, along with local-resolution maps, viewing direction plots, and gold-standard FSC plots, illustrating the resolution of the final maps generated from this data.

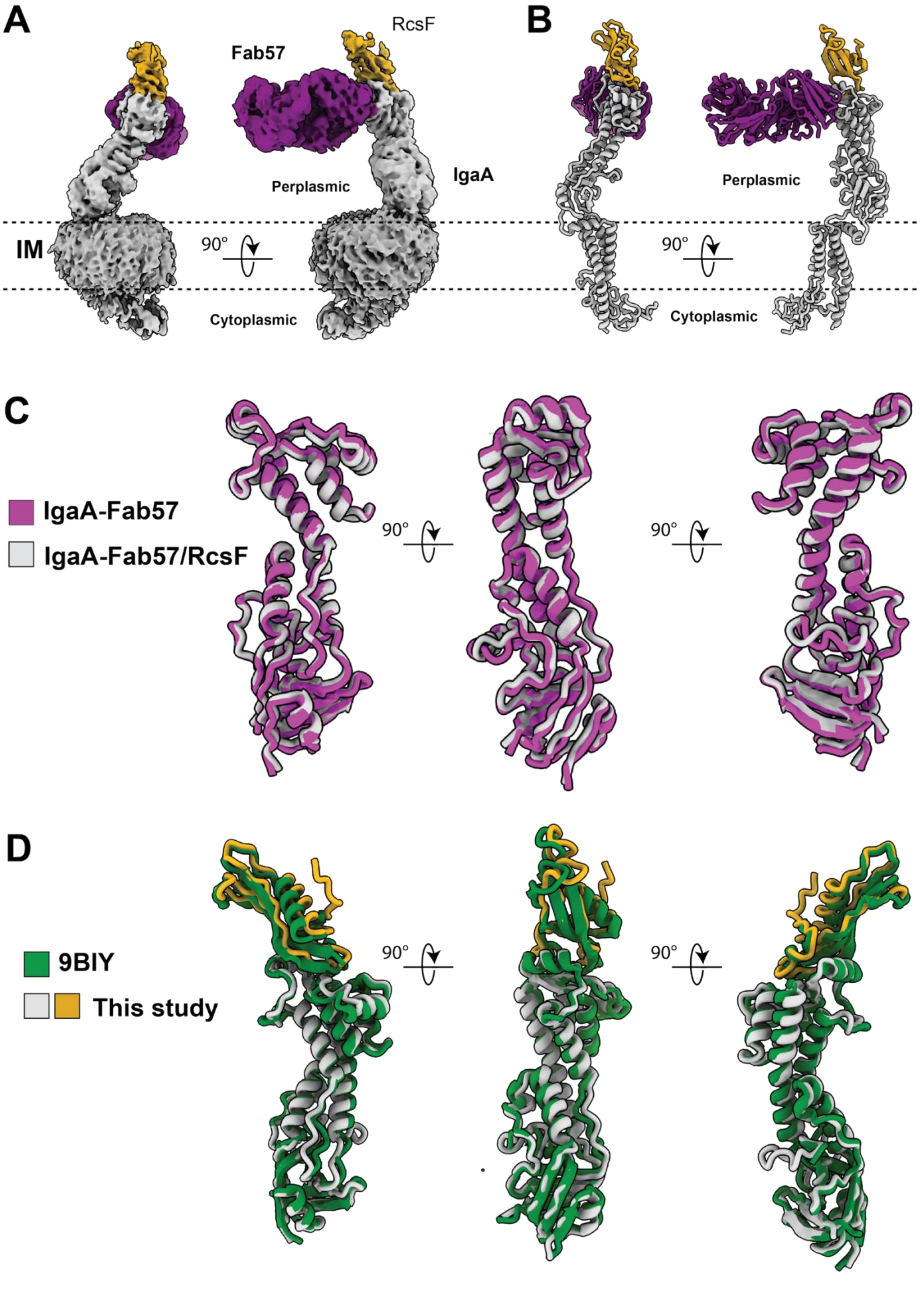
A
RcsF
Fab57
IgaA
Perplasmic
IM
90°
Cytoplasmic
B
Perplasmic
90°
Cytoplasmic
C
IgaA-Fab57
IgaA-Fab57/RcsF
90°
90°
D
9BIY
This study
90°
90°

Figure S7: Detailed views of the IgaA/Fab57/RcsF complex consisting of the full-length IgaA and structure comparison. A) Multiple views of maps of the full-length IgaA in complex with RcsF and Fab57. B) De novo built accurate structure of the IgaA/Fab57/RcsF complex into the map from panel A. C) Superposition of the Cryo-EM structure of IgaA/Fab57 with the IgaA/Fab57/RcsF complex; the overall rmsd is 0.9 Å. For clarity, Fab57 and RcsF have been removed. D) Superposition of the crystal structure of *E.coli* IgaA/RcsF (9BIY) with the IgaA/RcsF complex from this study; the overall rmsd is 1.3 Å.

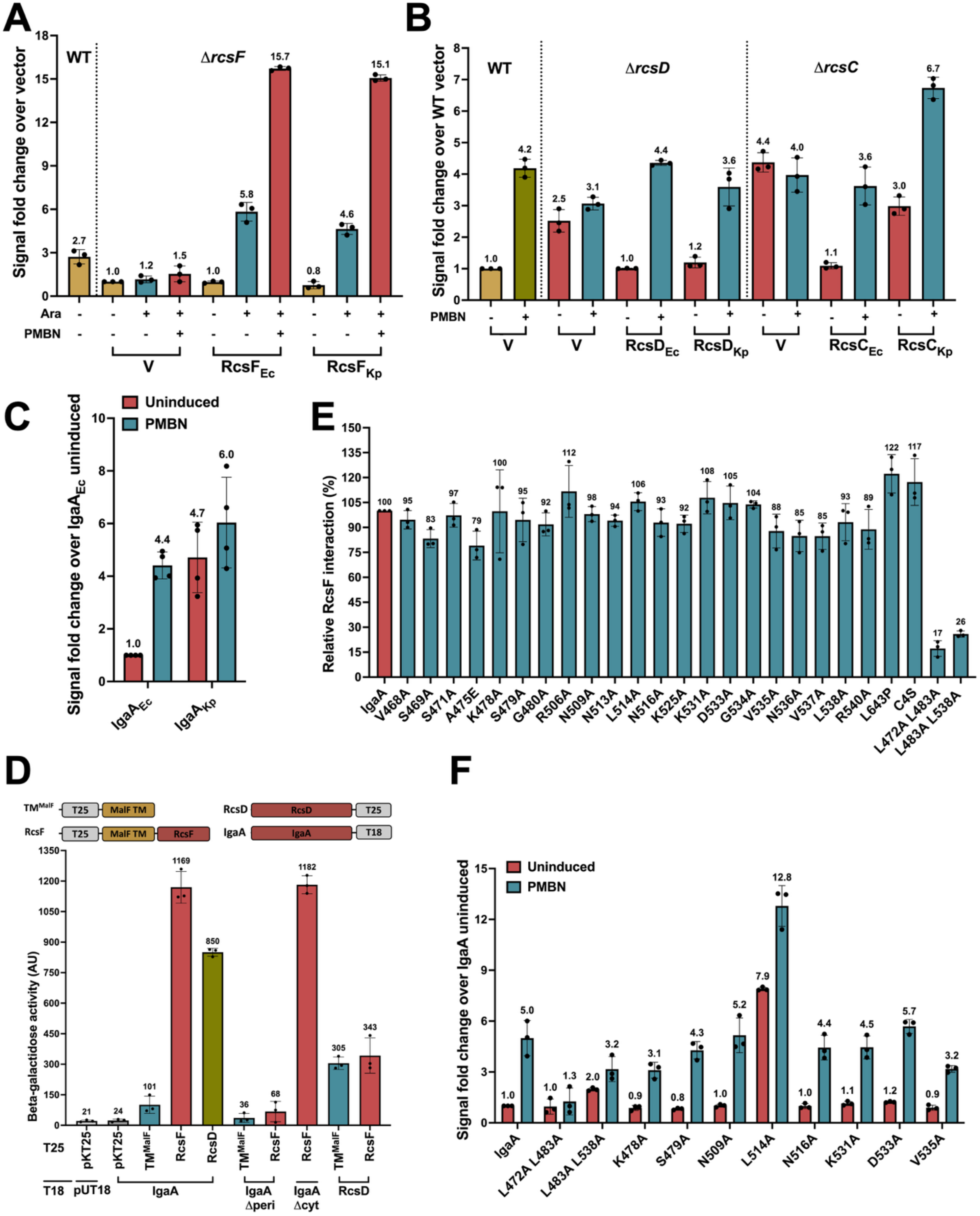

A
Signal fold change over vector
WT
ΔrcsF
2.7
1.0
1.2
1.5
1.0
5.8
15.7
0.8
4.6
15.1
Ara
PMBN
V
RcsFEc
RcsFKp
B
Signal fold change over WT vector
WT
ΔrcsD
ΔrcsC
1.0
4.2
2.5
3.1
1.0
4.4
1.2
3.6
4.4
4.0
1.1
3.6
3.0
6.7
PMBN
V
V
RcsDEc
RcsDKp
V
RcsCEc
RcsCKp
C
Uninduced
PMBN
Signal fold change over IgaAEc uninduced
1.0
4.4
4.7
6.0
IgaAEc
IgaAKp
E
Relative RcsF interaction (%)
100
95
83
97
79
100
95
92
112
98
94
106
93
92
108
105
104
88
85
85
93
89
122
117
17
26
IgaA
V468A
S469A
S471A
A475E
K478A
S479A
G480A
R506A
N509A
N513A
L514A
N516A
K525A
K531A
D533A
G534A
V535A
N536A
V537A
L538A
R540A
L643P
C4S
L472A L483A
L483A L538A
D
TMMalF
T25
MalF TM
RcsF
T25
MalF TM
RcsF
RcsD
RcsD
T25
IgaA
IgaA
T18
Beta-galactidose activity (AU)
21
24
101
1169
850
36
68
1182
305
343
T25
pKT25
pKT25
TMMalF
RcsF
RcsD
TMMalF
RcsF
RcsF
TMMalF
RcsF
T18
pUT18
IgaA
IgaA Δperi
IgaA Δcyt
RcsD
F
Uninduced
PMBN
Signal fold change over IgaA uninduced
1.0
5.0
1.0
1.3
2.0
3.2
0.9
3.1
0.8
4.3
1.0
5.2
7.9
12.8
1.0
4.4
1.1
4.5
1.2
5.7
0.9
3.2
IgaA
L472A L483A
L483A L538A
K478A
S479A
N509A
L514A
N516A
K531A
D533A
V535A

Figure S8: Measuring Rcs phosphorelay signaling and binding. A) Signaling by Klebsiella RcsF protein in *E. coli.* The strains WT (EAW8) and D*rcsF* (AP52) carry a *rprA* promoter fusion to mCherry (P_{rprA}::mCherry); mCherry fluorescence acts as a measure of Rcs activation. For the P_{rprA}::mCherry assay, the strains overexpressing pBAD33 (vector) or pBAD plasmids expressing $RcsF_{Ec}$ or $RcsF_{Kp}$ were grown in MOPS minimal glycerol medium containing chloramphenicol (25 mg/ml) and either 0.2% glucose or 0.01% arabinose at 37°C. The RFU at OD 0.4 compared to the uninduced (in the presence of glucose) D*rcsF* vector control (set to 1) is plotted. **B) Signaling by Klebsiella RcsD and RcsC proteins in *E. coli.*** The strain D*rcsD* (EAW19) overexpressing pBAD24 (vector), pBAD-$RcsD_{Ec}$ or $RcsD_{Kp}$ was grown in MOPS minimal glucose medium containing ampicillin (100 mg/ml) at 37°C. PMBN (20 mg/ml) was used for Rcs induction. The RFU at OD 0.4 compared to the uninduced WT vector control (set to 1) is plotted. The strain D*rcsC* (EAW91) overexpressing pBAD24 (vector), pBAD-$RcsC_{Ec}$ or $RcsC_{Kp}$ was assayed similarly. **C) Signaling by Klebsiella IgaA protein in *E. coli.*** The IgaA alleles are in the chromosome in a D*rcsD* strain carrying a P_{rpra}:mCherry reporter fusion. These strains with $IgaA_{Ec}$ (AP150) or $IgaA_{Kp}$ (AP149) were transformed with either pBAD24 vector (devoid of RcsD) or pEAW11 (*rcsD*$^{+}$) and their fluorescence assayed during growth in MOPS minimal glucose medium (with ampicillin). PMBN (20 µg/ml) was added in the medium at the beginning for Rcs activation. The RFU at OD 0.4 for each strain relative to the uninduced *igaA*+ *rcsD*$^{+}$ strain (set to 1) is plotted. **D.** BACTH for **interaction of *E. coli* Rcs proteins.** Beta-galactosidase activity was measured in a Δ*cyaA* strain (BTH101) expressing two plasmids encoding the T18 and T25 domains of adenylate cyclase fused to the proteins of interest, as shown at the top, and the expression measured compared to background. Note that the RcsF constructs all contain the MalF-TM domain in place of the usual RcsF lipoprotein domain. The IgaA/RcsD/RcsF protein fusion plasmids paired with their cognate vector had very low activity and were used as controls. **E. Interaction of IgaA mutants with RcsF**. The interaction of IgaA point mutants was tested with RcsF by BACTH in BTH101 as in S8E. **F. Signaling by IgaA mutant strains** Signaling was assayed with chromosomal *igaA* mutants, using the same method as Fig. S8C.

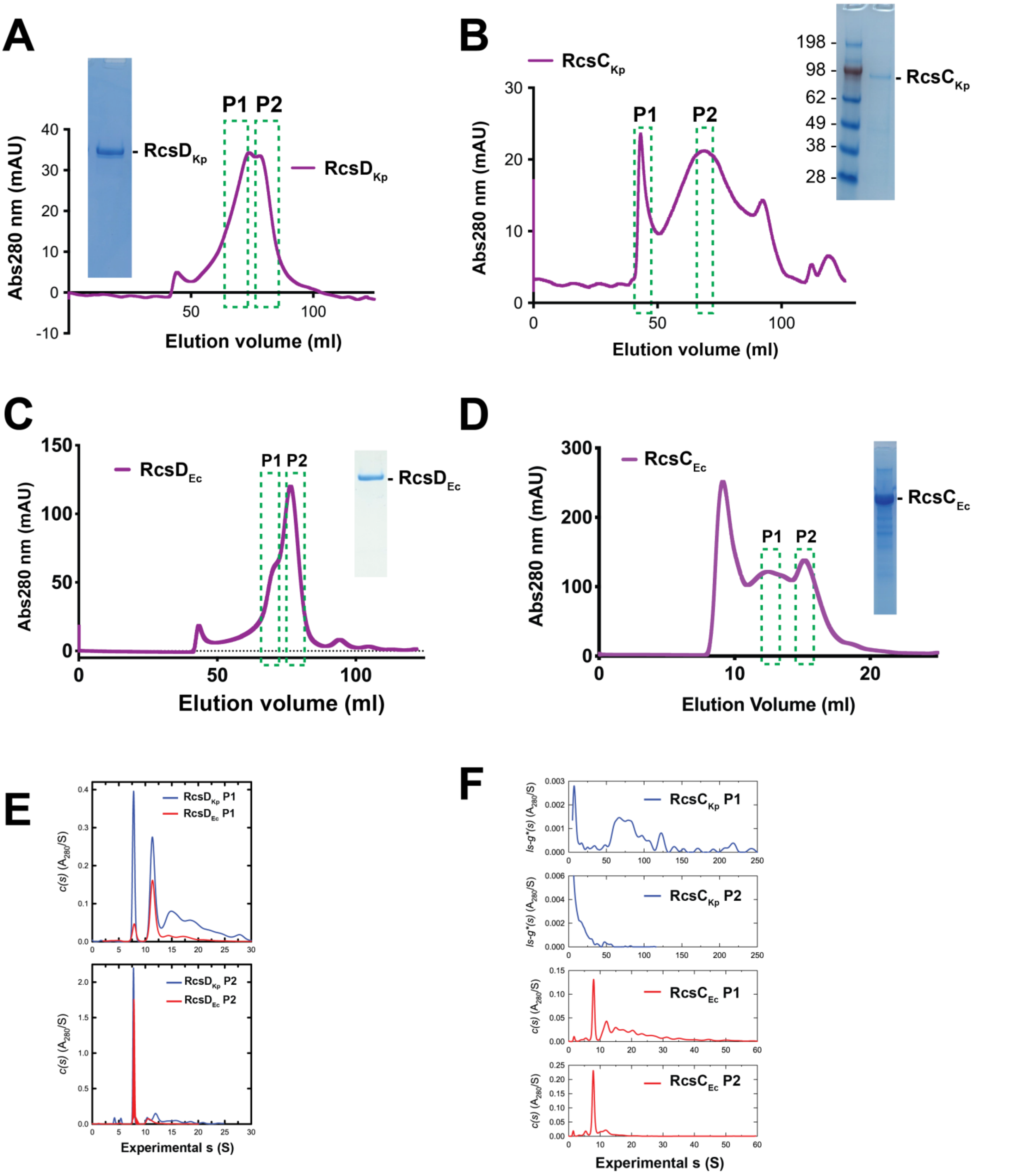

Figure S9: Biophysical studies of RcsD and RcsC. **A-D)** Size-exclusion chromatography (SEC) and SDS-PAGE gel analysis of purified RcsD and RcsC (from both *E. coli* and *K. pneumoniae*). Green boxes indicate the peaks/fractions used for AUC analysis. **E-F)** AUC results corresponding to panel A-D.

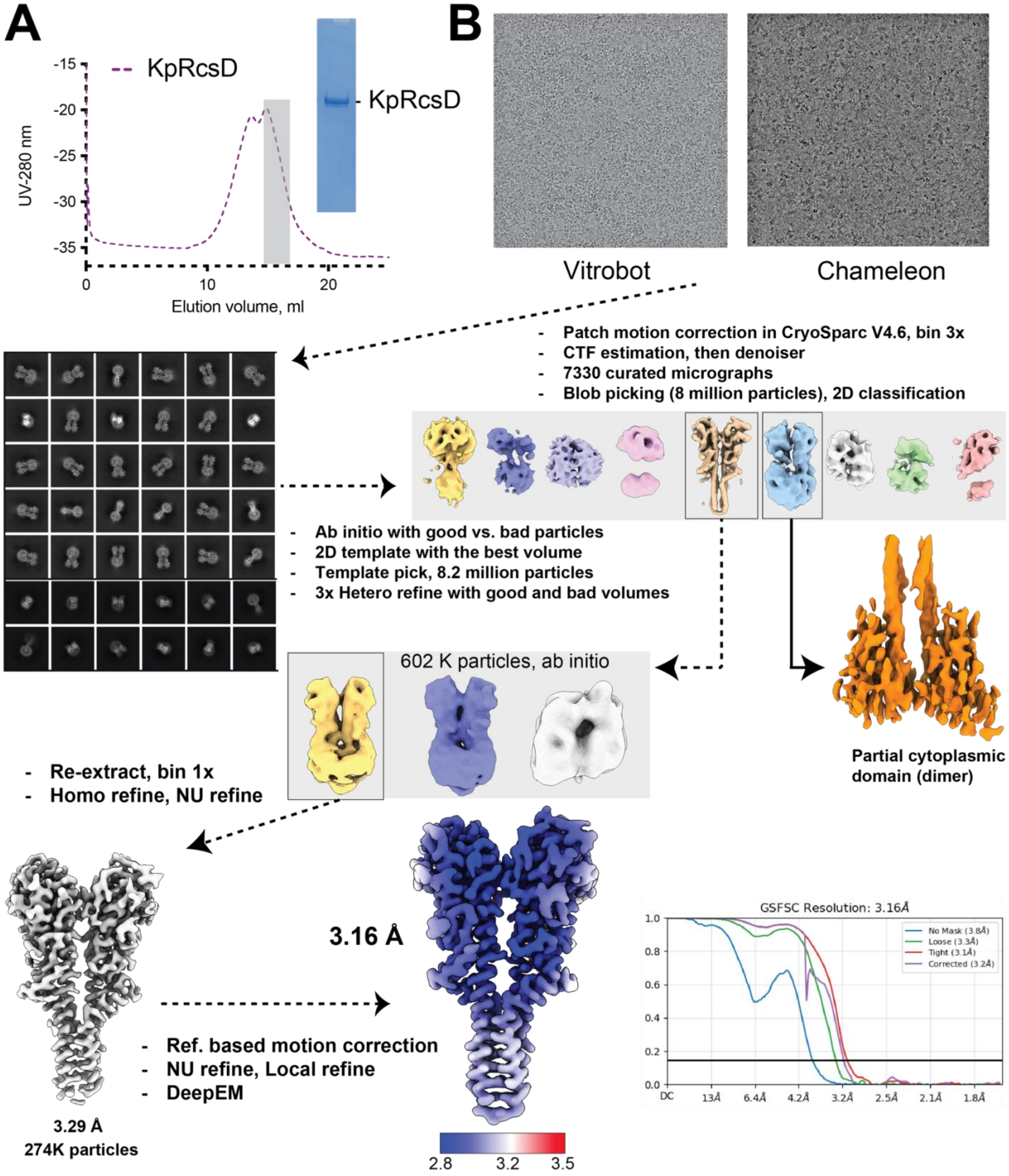

Figure S10: $RcsD_{Kp}$ data processing workflow. A) Chromatogram showing full-length RcsD purified by SEC column. The fractions used for structural studies are shaded gray. **B)** Schematic showing Cryo-EM data processing pipeline for RcsD. Note that only the TM/Periplasmic domains are visualized.

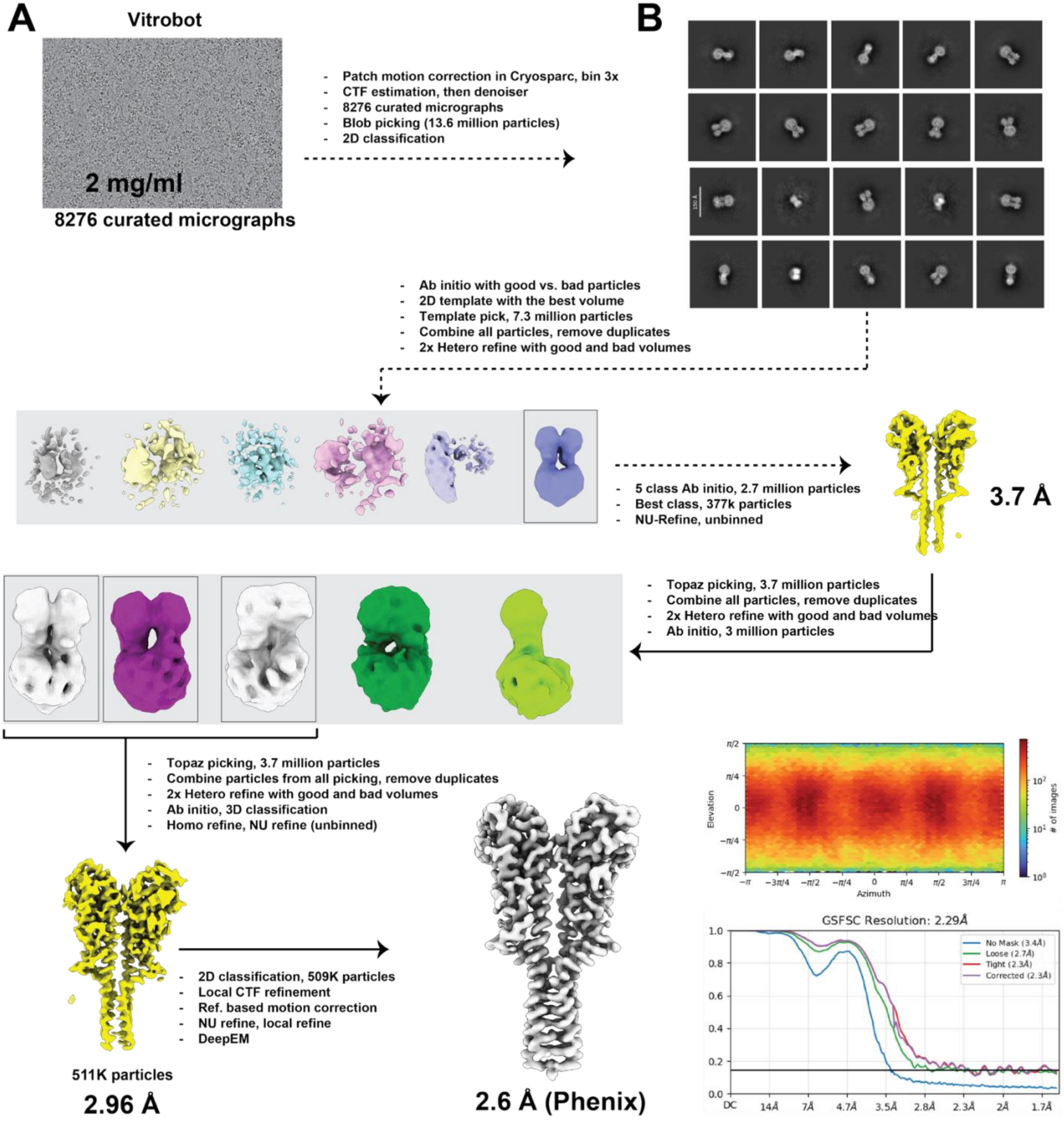

Figure S11: EcRcsD data processing workflow. A) Representative motion-corrected micrograph of EcRcsD particles. **B)** Examples of selected 2D class averages and schematic diagram of the Cryo-EM data-processing pipeline, viewing direction plot, and gold-standard FSC plot. Due to overfitting and resolution overestimation error in cryosparc, we are reporting the value calculated by Phenix, which is 2.6 Å.

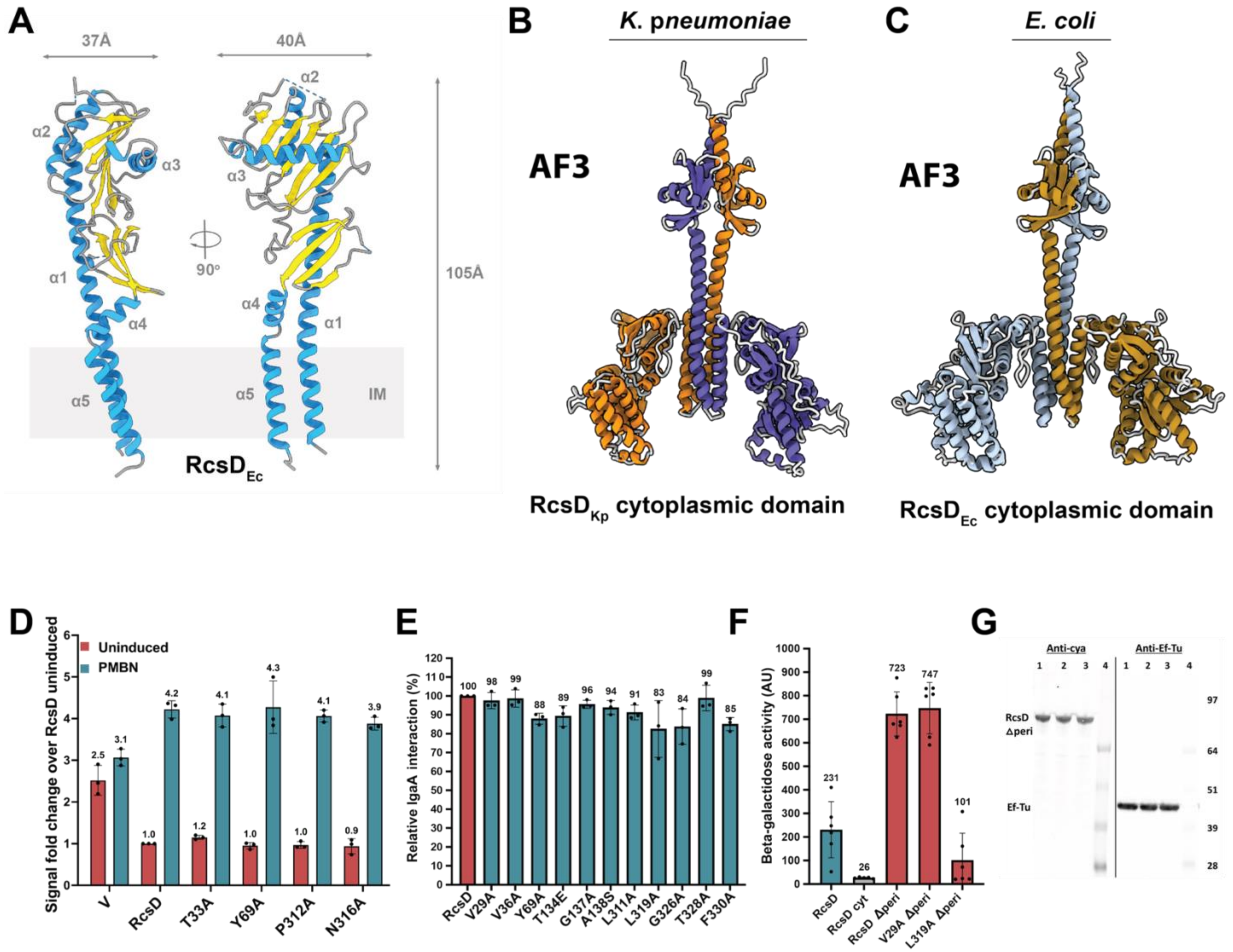

Figure S12: A) Domain organization of RcsD$_{Ec}$ (TM1/2 + Periplasmic, based on Cryo-EM structures)**. B-C)** AlphaFold3 predicted structures of cytoplasmic domains of RcsD homodimers from *K. pneumoniae* and *E. coli***. D)** Signaling by RcsD dimer interface mutants (assayed as in S8B). **E)** Interaction of RcsD mutants with IgaA. BACTH in BTH101 was used to measure the interaction of RcsD mutants with IgaA as in S8E. IgaA/RcsD interaction is set to 100 and other interactions are plotted relative to this. **F)** BACTH for RcsD homodimer formation. Homodimerization of various RcsD constructs was determined using BACTH in BTH101. **G)** Expression of RcsD mutants. Western blot of the T18 fusion constructs of RcsD Dperi, RcsD V29A Dperi, and RcsD L319A Dperi, expressed in a *cya*$^+$ strain (*DH5 alpha lacI$_Q$*), is depicted here. Samples were probed on parallel gels with either the anti-CyaA antibody or the anti Ef-Tu antibody (used as a control).

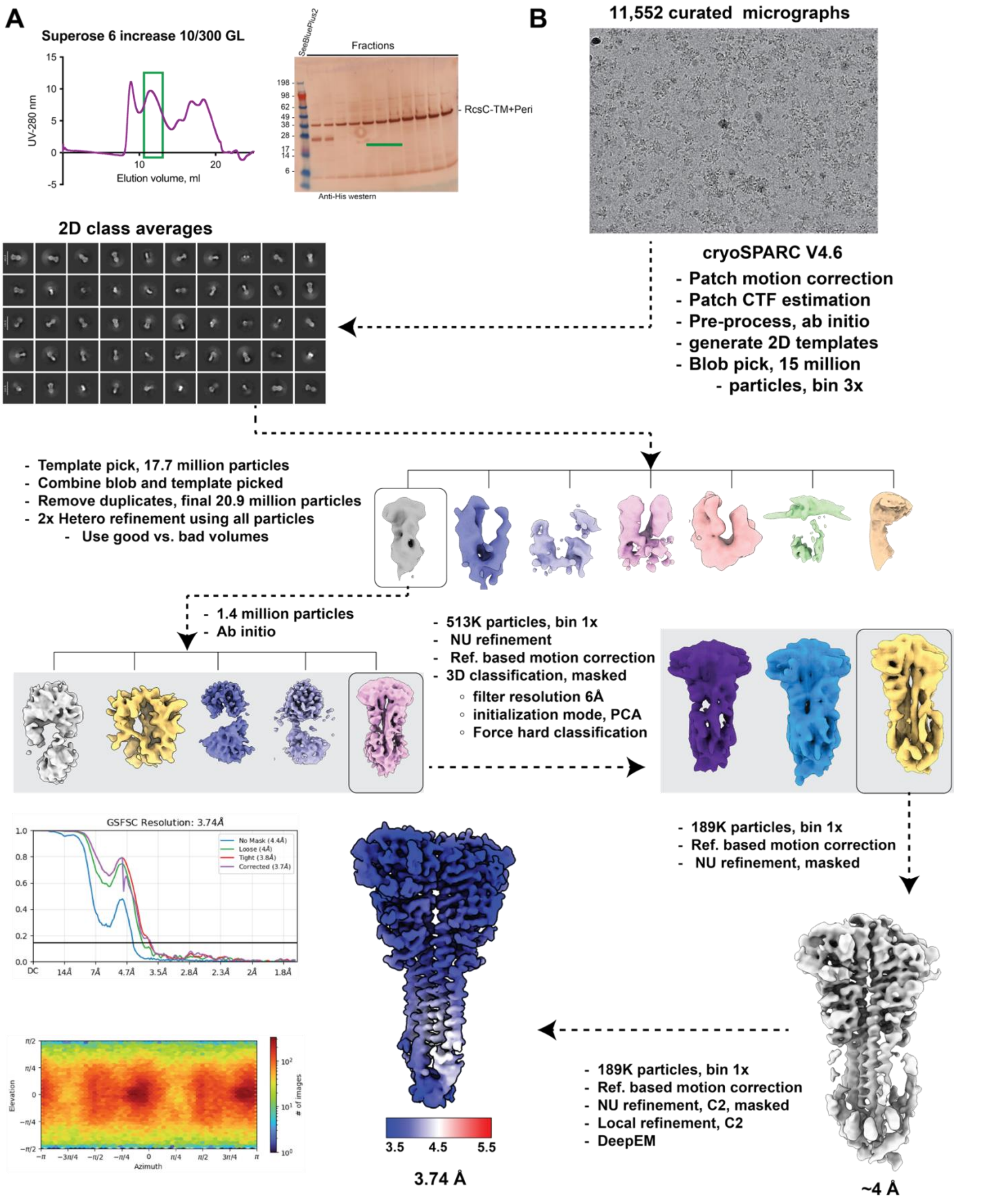

Figure S13: ***K. pneumoniae*** **RcsC data processing workflow.** A) Representative motion-corrected micrograph of $RcsC_{Kp}$ particles. B) Examples of selected 2D class averages and schematic diagram of the Cryo-EM data-processing pipeline, including local-resolution map, viewing direction plot, and gold-standard FSC plot.

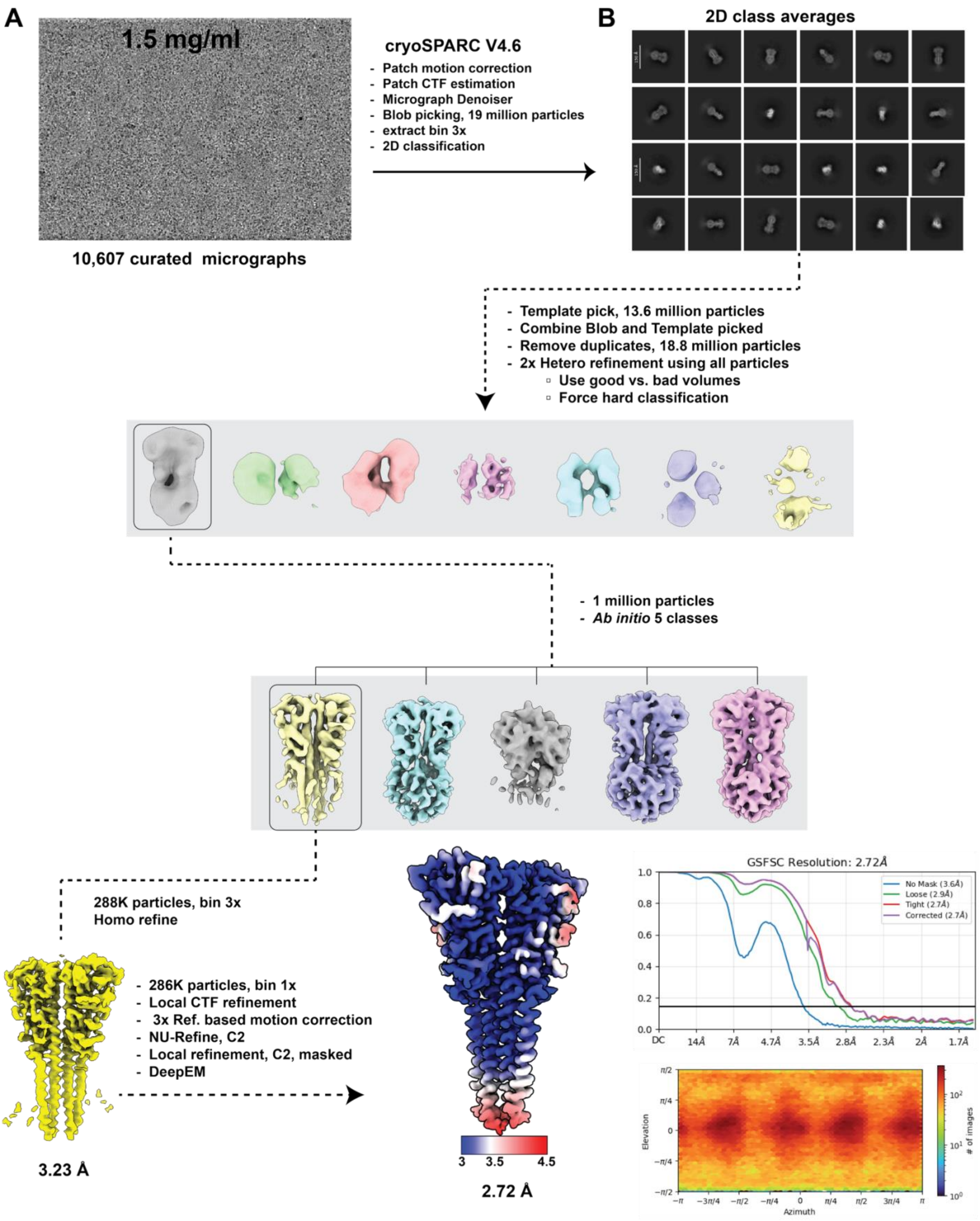

Figure S14: *E. coli* RcsC data processing workflow. A) Representative motion-corrected micrograph of RcsC$_{Ec}$ particles. B) Examples of selected 2D class averages and schematic diagram of the Cryo-EM data-processing pipeline, including local-resolution map, viewing direction plot, and gold-standard FSC plot.

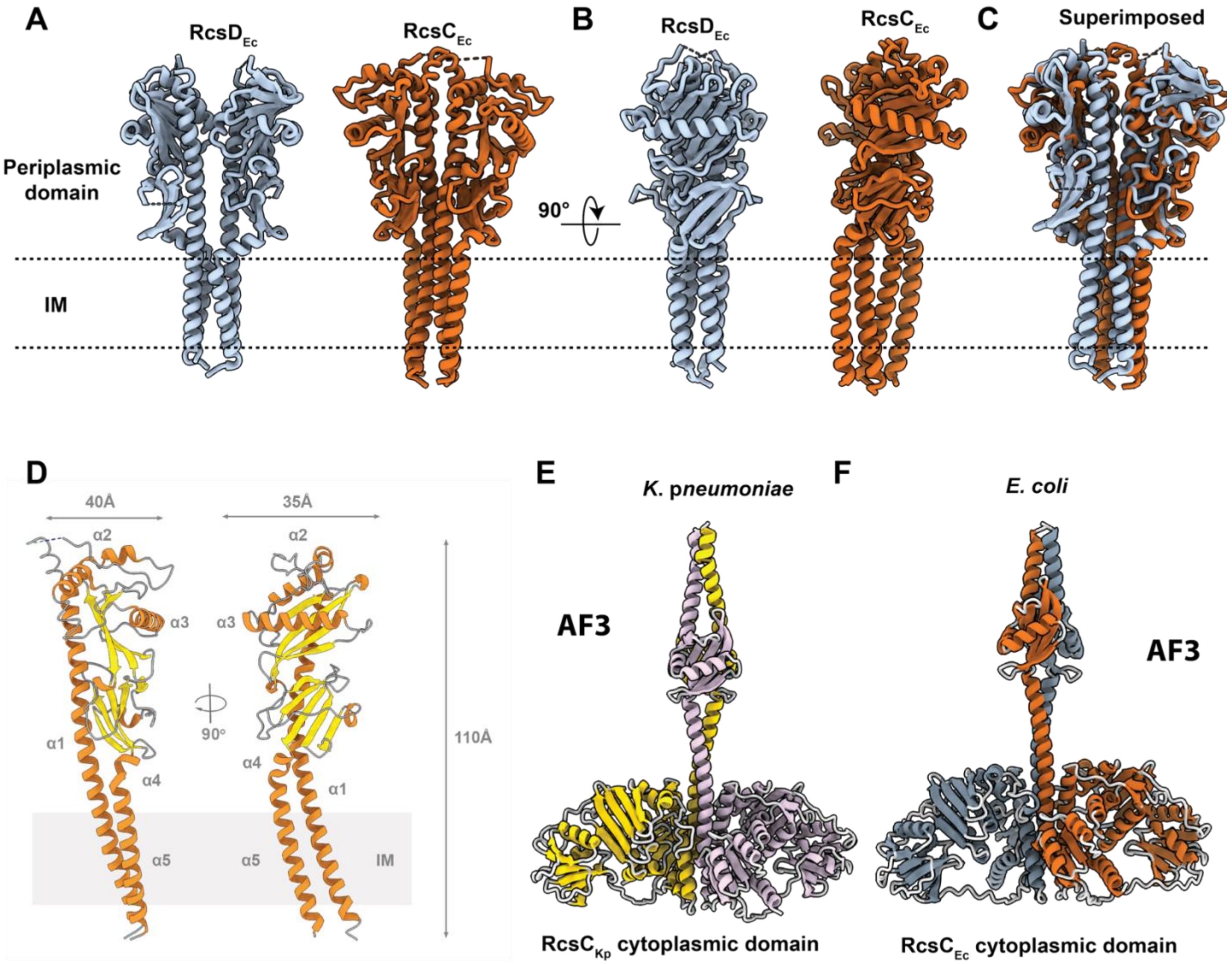

Figure S15: Comparison of RcsD and RcsC Structures. A-B) Side-by-side views of RcsD$_{Ec}$ and RcsC$_{Ec}$ Cryo-EM structures. **C)** Superposition of RcsD$_{Ec}$ and RcsC$_{Ec}$. **D)** Domain organization of RcsC$_{Ec}$ (TM1/2 + Periplasmic, based on Cryo-EM structures). **E-F)** AlphaFold3 predicted structures of cytoplasmic domains of RcsC homodimers from *K. pneumoniae* and *E. coli*.

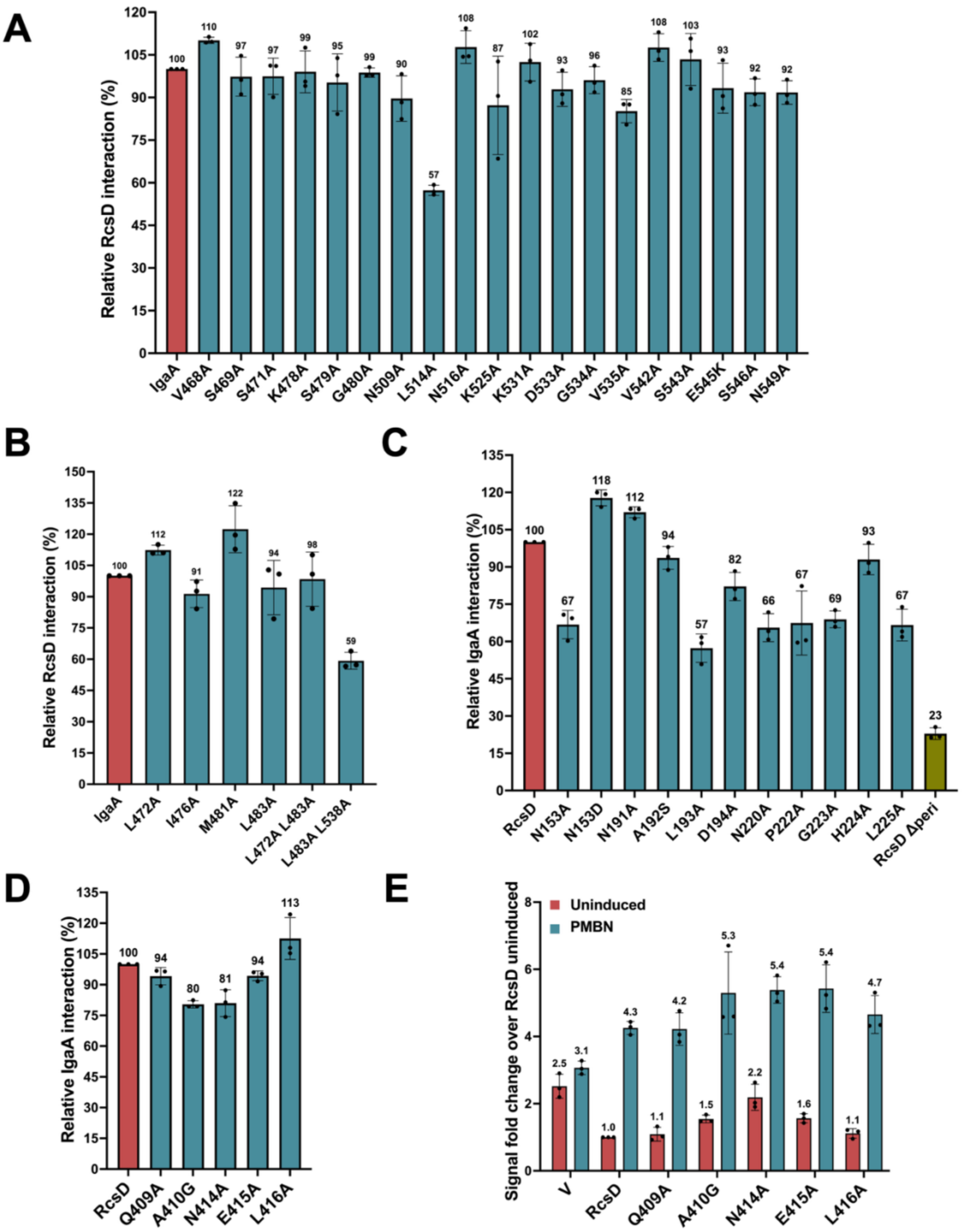

Figure S16: A) Interaction of IgaA mutants with RcsD. The interaction of RcsD was tested with IgaA and IgaA point mutants using BACTH as described in S8F. These IgaA mutants did not show a significant decrease in RcsD interaction. **B) Interaction of RcsF-defective IgaA mutants with RcsD.** The interaction of RcsD was tested with IgaA and IgaA point mutants in BTH101. These IgaA mutants have low interaction in RcsF but interact well with RcsD. **C) Interaction of RcsD periplasmic mutants with IgaA**. The binding of RcsD and its periplasmic point mutants to IgaA was tested using BACTH in BTH101. **D) Interaction of RcsD periplasmic mutants with IgaA**. The binding of RcsD and its periplasmic point mutants to IgaA was tested using BACTH in BTH101 as above. **E**) Signaling by RcsD cytoplasmic mutants. The D*rcsD* strain (EAW19) was transformed with either pBAD24 vector or RcsD mutants and their fluorescence assayed as described in S8B.

Supplementary Tables

Table S1: Cryo-EM data collection parameters, image processing and structure model building statistics

	$IgaA_{Kp}/RcsF_{Kp}$	Full-length $IgaA_{Kp}$/Fab57	Full-length $IgaA_{Kp}$-Fab57/$RcsF_{Kp}$
**EMDB-ID**	75234	75236	75238
**Data collection**			
Microscope	Glacios	Titan Krios	Titan Krios
Voltage (kV)	200	300	300
Magnification	45000x	45000x	105,000x
Pixel size (Å)	0.89	0.86	0.824
Electron dose (e^-/Å^2)	65.45-70.43	60.24	46-58
Number of frames	32	28	40
Defocus range	-0.8 - 2.4	-0.8 - 2.4	-0.8 - 2.8
**Image processing**			
Micrographs selected (#)	7,619	7,457	11,930
Total extracted particles (#)	11.4	13 mil	22 mil
Final particles used (#)	209K	377K	460K
Symmetry imposed	C1	C1	C1
Map resolution, FSC 0.143 (Å)	5.44	5.5	2.88
Map sharpening *B*-factor (Å^2)	-341.4	-156.8	-85.1

Table S2: Cryo-EM data collection parameters, image processing and structure model building statistics

	$IgaA_{Kp}$/Fab57	$IgaA_{Kp}$-Fab57/$RcsF_{Kp}$	$RcsD_{Kp}$	$RcsD_{Ec}$	$RcsC_{Kp}$	$RcsC_{Ec}$
**EMDB-ID**	75235	75237	75239	75240	75241	75242
**PDB-ID**	10KG	10KH	10KI	10KJ	10KK	10KL
**Data collection**						
Microscope	Titan Krios	Titan Krios	Titan Krios	Titan Krios	Titan Krios	Titan Krios
Voltage (kV)	300	300	300	300	300	300
Magnification	45,000x	105,000x	105,000x	105,000x	105,000x	105,000x
Pixel size (Å)	0.86	0.824	0.858	0.824	0.858	0.824
Electron dose (e^-/Å^2)	60.24	46-58	60	59.12	61	55.26
Number of frames	28	40	40 and 50	40	40	40
Defocus range	-0.8 - 2.4	-0.8 - 2.8	-0.8 - 2.2	-0.8 - 2.2	-0.8 - 2.6	-0.8 - 2.6
**Image processing**						
Micrographs selected (#)	7,457	11,930	7,330	8,276	11,552	10,607
Total extracted particles (#)	13 mil	22 mil	8.2 mil	13.6 mil	15 mil	19 mil

Final particles used (#)	489K	307K	274K	511K	189K	286K
Symmetry imposed	C1	C1	C1	C2	C2	C2
Map resolution, FSC 0.143 (Å)	2.87	2.79	3.16	2.6	3.74	2.72
Map sharpening *B*-factor (Å^2)	-102.7	-87.5	-143.2	-70.5	-117.6	-91.8
**Model Refinement & validation**						
r.m.s.d. Bond lengths (Å)	0.006	0.004	0.004	0.009	0.007	0.005
r.m.s.d. Bond angles (°)	0.835	0.588	0.695	0.981	0.912	0.838
Ramachandran Favored (%)	95.2	97.74	97.81	95.27	93.29	97.97
Ramachandran Allowed (%)	4.8	2.26	2.91	4.73	6.71	2.03
Ramachandran Disallowed (%)	0.0	0.0	0.0	0.0	0.0	0.0
Clash score	2.42	8.02	7.17	5.35	6.33	3.93
Rotamer outliers (%)	0.0	0.0	0.0	0.0	0.0	0.0
Map CC (volume)	0.9	0.84	0.85	0.8	0.73	0.81
**Model Composition**						
Protein residues	715	805	642	604	690	648

Table S3: List of strains used in this study

Name	Genotype	Method of construction or reference
MG1655	Wild-type *E. coli* K-12	Lab collection
BTH101	*F-, cya-99, araD139, galE15, galK16, rpsL1 (Str^r), hsdR2, mcrA1, mcrB1*	Karimova et al., 1998[2]
NEB DH5-alpha F'IQ	F′ *proA*$^+$*B*$^+$ *lacI*^{q} Δ*(lacZ)M15 zzf::Tn10* (Tet^R) / *fhuA2*Δ*(argF-lacZ)U169 phoA glnV44 Φ80Δ(lacZ)M15 gyrA96 recA1 relA1 endA1 thi-1 hsdR17*	New England Biolabs
EAW8	Δ*araBAD*::P$_{rprA142}$-mCherry, Δ*araE*p P$_{CP6}$::*gent::*P$_{cp18}$-*araE*	Wall et al., 2020[3]
EAW12	*rcsD*541(::FRT), *cya*	Wall et al., 2020[3]
EAW19	Δ*araBAD*::P$_{rprA142}$-mCherry, *rcsD*541(::FRT), Δ*araEp* P$_{cp6}$*gent::*P$_{cp18}$-*araE*	Wall et al., 2020[3]
EAW90	Δ*araBAD*::P$_{rprA142}$-mCherry, Δ*araE*p P$_{cp6}$*gent*::P$_{cp18}$-*araE*, *rcsD541(::FRT)*, Δ*igaA*::*kan-araC-kid*	Wall et al., 2020[3]
EAW91	Δ*araBAD*::P$_{rprA142}$-mCherry, Δ*araEp* P$_{cp6}$*gent::*P$_{cp18}$-*araE,* Δ*rcsC91*	Wall et al., 2020[3]
AP24	Δ*araBAD*::P$_{rprA142}$-mCherry, Δ*araEp* P$_{cp6}$*gent::*P$_{cp18}$-*araE, rcsD*541(::FRT), Δ*rcsF*	EAW19 + P1 (Keio JW0192)
AP27	Δ*araBAD*::P$_{rprA142}$-mCherry, Δ*araE*p P$_{cp6}$*gent*::P$_{cp18}$-*araE*, *rcsD541(::FRT)*, Δ*rcsF, igaA N513A*	AP184 + P1 (Keio JW0192)
AP29	Δ*araBAD*::P$_{rprA142}$-mCherry, Δ*araE*p P$_{cp6}$*gent*::P$_{cp18}$-*araE*, *rcsD541(::FRT)*, Δ*rcsF, igaA R540A*	AP216 + P1 (Keio JW0192)
AP51	Δ*araBAD*::P$_{rprA142}$-mCherry, Δ*araE*p P$_{CP6}$::*gent::*P$_{cp18}$-*araE, rcsF::kan*	Petchiappan et al., 2024[4]
AP52	Δ*araBAD*::P$_{rprA142}$-mCherry, Δ*araE*p P$_{CP6}$::*gent::*P$_{cp18}$-*araE,* Δ*rcsF*	AP51 + pCP20
AP140	Δ*araBAD*::P$_{rprA142}$-mCherry, Δ*araE*p P$_{cp6}$*gent*::P$_{cp18}$-*araE*, *rcsD541(::FRT)*, *igaA L438A L538A*	AP179 recombination with PCR product of oligos EAW213 and EAW214 on pAP105382 template
AP141	Δ*araBAD*::P$_{rprA142}$-mCherry, Δ*araE*p P$_{cp6}$*gent*::P$_{cp18}$-*araE*, *rcsD541(::FRT)*, *igaA A94D*	AP179 recombination with PCR product of oligos EAW213 and EAW214 on pAP1094 template

AP142	Δ*araBAD*::$P_{rprA142}$-mCherry, Δ*araE*p P_{cp6}*gent*::P_{cp18}-*araE*, *rcsD541(::FRT)*, *igaA I476A*	AP179 recombination with PCR product of oligos EAW213 and EAW214 on pAP10476 template
AP144	Δ*araBAD*::$P_{rprA142}$-mCherry, Δ*araE*p P_{cp6}*gent*::P_{cp18}-*araE*, *rcsD541(::FRT)*, *igaA L483A*	AP179 recombination with PCR product of oligos EAW213 and EAW214 on pAP10483 template
AP145	Δ*araBAD*::$P_{rprA142}$-mCherry, Δ*araE*p P_{cp6}*gent*::P_{cp18}-*araE*, *rcsD541(::FRT)*, *igaA V535A*	AP179 recombination with PCR product of oligos EAW213 and EAW214 on pAP10535 template
AP146	Δ*araBAD*::$P_{rprA142}$-mCherry, Δ*araE*p P_{cp6}*gent*::P_{cp18}-*araE*, *rcsD541(::FRT)*, *igaA N536A*	AP179 recombination with PCR product of oligos EAW213 and EAW214 on pAP10536 template
AP147	Δ*araBAD*::$P_{rprA142}$-mCherry, Δ*araE*p P_{cp6}*gent*::P_{cp18}-*araE*, *rcsD541(::FRT)*, *igaA V537A*	AP179 recombination with PCR product of oligos EAW213 and EAW214 on pAP10537 template
AP148	Δ*araBAD*::$P_{rprA142}$-mCherry, Δ*araE*p P_{cp6}*gent*::P_{cp18}-*araE*, *rcsD541(::FRT)*, *igaA L538A*	AP179 recombination with PCR product of oligos EAW213 and EAW214 on pAP10538 template
AP149	Δ*araBAD*::$P_{rprA142}$-mCherry, Δ*araE*p P_{cp6}*gent*::P_{cp18}-*araE*, *rcsD541(::FRT)*, $igaA_{Kp}$	AP179 recombination with PCR product of oligos EAW213 and EAW214 on pAP104 template
AP150	Δ*araBAD*::$P_{rprA142}$-mCherry, Δ*araE*p P_{cp6}*gent*::P_{cp18}-*araE*, *rcsD541(::FRT)*, *igaA*	AP179 recombination with PCR product of oligos EAW213 and EAW214 on pEAW1 template
AP151	Δ*araBAD*::$P_{rprA142}$-mCherry, Δ*araE*p P_{cp6}*gent*::P_{cp18}-*araE*, *rcsD541(::FRT)*, *igaA R97A*	AP179 recombination with PCR product of oligos EAW213 and EAW214 on pAP1097 template
AP152	Δ*araBAD*::$P_{rprA142}$-mCherry, Δ*araE*p P_{cp6}*gent*::P_{cp18}-*araE*, *rcsD541(::FRT)*, *igaA R97S*	AP179 recombination with PCR product of oligos EAW213 and EAW214 on pAP10972 template
AP153	Δ*araBAD*::$P_{rprA142}$-mCherry, Δ*araE*p P_{cp6}*gent*::P_{cp18}-*araE*, *rcsD541(::FRT)*, *igaA R506A*	AP179 recombination with PCR product of oligos EAW213 and EAW214 on pAP10506 template
AP179	Δ*araBAD*::$P_{rprA142}$-mCherry, Δ*araE*p P_{cp6}*gent*::P_{cp18}-*araE*, *rcsD541(::FRT)*, Δ*igaA*::*kan-araC-kid,* pSIM6	EAW90 + pSIM6
AP183	Δ*araBAD*::$P_{rprA142}$-mCherry, Δ*araE*p P_{cp6}*gent*::P_{cp18}-*araE*, *rcsD541(::FRT)*, *igaA M481A*	AP179 recombination with PCR product of oligos EAW213 and EAW214 on pAP10481 template
AP184	Δ*araBAD*::$P_{rprA142}$-mCherry, Δ*araE*p P_{cp6}*gent*::P_{cp18}-*araE*, *rcsD541(::FRT)*, *igaA N513A*	AP179 recombination with PCR product of oligos EAW213 and EAW214 on pAP10513 template

AP185	Δ*araBAD*::$P_{rprA142}$-mCherry, Δ*araE*p P_{cp6}*gent*::P_{cp18}-*araE*, *rcsD541(::FRT)*, *igaA L472A*	AP179 recombination with PCR product of oligos EAW213 and EAW214 on pAP10472 template
AP186	Δ*araBAD*::$P_{rprA142}$-mCherry, Δ*araE*p P_{cp6}*gent*::P_{cp18}-*araE*, *rcsD541(::FRT)*, *igaA K478A*	AP179 recombination with PCR product of oligos EAW213 and EAW214 on pAP10478 template
AP188	Δ*araBAD*::$P_{rprA142}$-mCherry, Δ*araE*p P_{cp6}*gent*::P_{cp18}-*araE*, *rcsD541(::FRT)*, *igaA S479A*	AP179 recombination with PCR product of oligos EAW213 and EAW214 on pAP10479 template
AP215	Δ*araBAD*::$P_{rprA142}$-mCherry, Δ*araE*p P_{cp6}*gent*::P_{cp18}-*araE*, *rcsD541(::FRT)*, *igaA L472A L483A*	AP179 recombination with PCR product of oligos EAW213 and EAW214 on pAP104722 template
AP216	Δ*araBAD*::$P_{rprA142}$-mCherry, Δ*araE*p P_{cp6}*gent*::P_{cp18}-*araE*, *rcsD541(::FRT)*, *igaA R540A*	AP179 recombination with PCR product of oligos EAW213 and EAW214 on pAP10540 template
AP233	Δ*araBAD*::$P_{rprA142}$-mCherry, Δ*araE*p P_{cp6}*gent*::P_{cp18}-*araE*, *rcsD541(::FRT)*, *igaA N509A*	AP179 recombination with PCR product of oligos EAW213 and EAW214 on pAP10509 template
AP234	Δ*araBAD*::$P_{rprA142}$-mCherry, Δ*araE*p P_{cp6}*gent*::P_{cp18}-*araE*, *rcsD541(::FRT)*, *igaA L514A*	AP179 recombination with PCR product of oligos EAW213 and EAW214 on pAP10514 template
AP235	Δ*araBAD*::$P_{rprA142}$-mCherry, Δ*araE*p P_{cp6}*gent*::P_{cp18}-*araE*, *rcsD541(::FRT)*, *igaA N516A*	AP179 recombination with PCR product of oligos EAW213 and EAW214 on pAP10516 template
AP236	Δ*araBAD*::$P_{rprA142}$-mCherry, Δ*araE*p P_{cp6}*gent*::P_{cp18}-*araE*, *rcsD541(::FRT)*, *igaA K531A*	AP179 recombination with PCR product of oligos EAW213 and EAW214 on pAP10531 template
AP237	Δ*araBAD*::$P_{rprA142}$-mCherry, Δ*araE*p P_{cp6}*gent*::P_{cp18}-*araE*, *rcsD541(::FRT)*, *igaA D533A*	AP179 recombination with PCR product of oligos EAW213 and EAW214 on pAP10533 template

Table S4: List of plasmids used in this study

Name	Description	Method of construction/ Reference
pBAD24	pBR322-based vector for protein expression driven by the *araBAD* operon promoter (arabinose inducible) (Amp^r)	Guzman et al., 1995[5]
pBAD33	pACYC184-based vector for protein expression driven by the *araBAD* operon promoter (arabinose inducible) (Chl^r)	Guzman et al., 1995[5]
pCP20	Plasmid with temperature-sensitive origin of replication, encoding the FLP recombinase	Cherepanov et al., 1995[6]
pUT18	Vector encoding the Cya T18 fragment under lac promoter control (Amp^r)	Karimova et al., 2001[7]
pKT25	Vector encoding the Cya T25 fragment under lac promoter control (Kan^r)	Karimova et al., 2001[7]
pKNT25	Vector encoding the Cya T25 fragment under lac promoter control (Kan^r)	Karimova et al., 2005[8]
pSIM6	Plasmid with temperature-sensitive origin of replication, encoding l-Red cI857, gam-beta-exo (Amp^r)	https://ncifrederick.cancer.gov/ recombineering/strains-plasmids-and-primers
pSIM27	Plasmid with temperature-sensitive origin of replication, encoding l-Red cI857, gam-beta-exo (Tet^r)	https://ncifrederick.cancer.gov/ recombineering/strains-plasmids-and-primers
pBAD28	pBAD18, pACYC184 vector for protein expression driven by the *araBAD* operon promoter (arabinose inducible), (Amp^r)	Guzman et al., 1995[5]
pET28	T7 promoter based for high-level protein expression, Kanamycin (Kan^r)	
**RcsF plasmids:**		
pAP3330	$RcsF_{Kp}$ cloned in pBAD33 vector	pBAD33 linearized with primers AP559 and AP560; Insert is gBlock AP_GF33kp
pAP3340	RcsF cloned in pBAD33 vector	Petchiappan et al., 2024
pAP405	MalF TM-RcsF ($MalF_{1\text{-}38}$-$RcsF_{17\text{-}C}$) with T25 tag at N-terminal cloned in pKT25	pKT25 linearized with EW3 and EW4; Insert is gBlock AP_FMF25
pAP406	RcsF A55K (with MalF TM at N-terminal) with T25 tag in pKT25	pAP405 template with primers AP561 and AP562 (SDM)
pAP411	RcsF A55V (with MalF TM at N-terminal) with T25 tag in pKT25	pAP405 template with primers AP773 and AP774 (SDM)

pAP412	RcsF L58Y (with MalF TM at N-terminal) with T25 tag in pKT25	pAP405 template with primers AP775 and AP776 (SDM)
pAP413	MalF TM helix with T25 tag at N-terminal cloned in pKT25	pAP405 template with primers AP397and EW4
pAP415	RcsF P62L (with MalF TM at N-terminal) with T25 tag in pKT25	pAP405 template with primers AP819and AP 820 (SDM)
pAP417	RcsF K61A (with MalF TM at N-terminal) with T25 tag in pKT25	pAP405 template with primers AP903 and AP904 (SDM)
pAP418	RcsF F63A (with MalF TM at N-terminal) with T25 tag in pKT25	pAP405 template with primers AP905 and AP906 (SDM)
pAP419	RcsF R64A (with MalF TM at N-terminal) with T25 tag in pKT25	pAP405 template with primers AP907and AP908 (SDM)
**IgaA plasmids:**		
pEAW1	IgaA with T18 tag at C-terminal cloned in pUT18	Wall et al., 2020[3]
pEAW1C4S	IgaA C404S C425S C498S C504S (C4S)-T18 tag in pUT18	Petchiappan et al., 2024[4]
pEAW1L	IgaA L643P- T18 tag in pUT18	Wall et al., 2020[3]
pEAW1peri	IgaA Δperi$_{384-649}$ with T18 tag at C-terminal cloned in pUT18	Wall et al., 2020[3]
pEAW2	IgaA with T25 tag at C-terminal cloned in pKNT25	Wall et al., 2020[3]
pAP101	IgaA with cyt1 and cyt2 domains deleted (Δ36-181 Δ263-329) -T18 tag at C-terminal cloned in pUT18	Petchiappan et al., 2024[4]
pAP1094	IgaA A94D – T18 tag in pUT18	pEAW1 template with primers AP553 and AP554 (SDM)
pAP1097	IgaA R97A– T18 tag in pUT18	pEAW1 template with primers AP143 and AP144 (SDM)
pAP10972	IgaA R97S– T18 tag in pUT18	pEAW1 template with primers AP681 and AP682 (SDM)
pAP10468	IgaA V468A– T18 tag in pUT18	pEAW1 template with primers AP827 and AP828 (SDM)
pAP10469	IgaA S469A– T18 tag in pUT18	pEAW1 template with primers AP637 and AP638 (SDM)
pAP10471	IgaA S471A– T18 tag in pUT18	pEAW1 template with primers AP469 and AP470 (SDM)
pAP10472	IgaA L472A– T18 tag in pUT18	pEAW1 template with primers AP707 and AP708 (SDM)
pAP10475	IgaA A475E– T18 tag in pUT18	pEAW1 template with primers AP423 and AP424 (SDM)
pAP10476	IgaA I476A– T18 tag in pUT18	pEAW1 template with primers AP779 and AP780 (SDM)

pAP10478	IgaA K478A– T18 tag in pUT18	pEAW1 template with primers AP735 and AP736 (SDM)
pAP10479	IgaA S479A– T18 tag in pUT18	pEAW1 template with primers AP639 and AP640 (SDM)
pAP10480	IgaA G480A– T18 tag in pUT18	pEAW1 template with primers AP709 and AP710 (SDM)
pAP10481	IgaA M481A– T18 tag in pUT18	pEAW1 template with primers AP711 and AP712 (SDM)
pAP10483	IgaA L483A– T18 tag in pUT18	pEAW1 template with primers AP809 and AP810 (SDM)
pAP10506	IgaA R506A– T18 tag in pUT18	pEAW1 template with primers AP589 and AP590 (SDM)
pAP10509	IgaA N509A– T18 tag in pUT18	pEAW1 template with primers AP299 and AP300 (SDM)
pAP10513	IgaA N513A– T18 tag in pUT18	pEAW1 template with primers AP591 and AP592 (SDM)
pAP10514	IgaA L514A– T18 tag in pUT18	pEAW1 template with primers AP939 and AP940 (SDM)
pAP10516	IgaA N516A– T18 tag in pUT18	pEAW1 template with primers AP813 and AP814 (SDM)
pAP10525	IgaA K525A– T18 tag in pUT18	pEAW1 template with primers AP713 and AP714 (SDM)
pAP10531	IgaA K531A– T18 tag in pUT18	pEAW1 template with primers AP783 and AP784 (SDM)
pAP10533	IgaA D533A– T18 tag in pUT18	pEAW1 template with primers AP785 and AP786 (SDM)
pAP10534	IgaA G534A– T18 tag in pUT18	pEAW1 template with primers AP891 and AP892 (SDM)
pAP10535	IgaA V535A– T18 tag in pUT18	pEAW1 template with primers AP787 and AP788 (SDM)
pAP10536	IgaA N536A– T18 tag in pUT18	pEAW1 template with primers AP415 and AP416 (SDM)
pAP10537	IgaA V537A– T18 tag in pUT18	pEAW1 template with primers AP789 and AP790 (SDM)
pAP10538	IgaA L538A– T18 tag in pUT18	pEAW1 template with primers AP791 and AP792 (SDM)
pAP10540	IgaA R540A– T18 tag in pUT18	pEAW1 template with primers AP815 and AP816 (SDM)
pAP10542	IgaA V542A– T18 tag in pUT18	pEAW1 template with primers AP301 and AP302 (SDM)
pAP10543	IgaA S543A– T18 tag in pUT18	pEAW1 template with primers AP303 and AP304 (SDM)
pAP10545	IgaA E545K– T18 tag in pUT18	pEAW1 template with primers AP185 and AP186 (SDM)

pAP10546	IgaA S546A– T18 tag in pUT18	pEAW1 template with primers AP305 and AP306 (SDM)
pAP10549	IgaA N549A– T18 tag in pUT18	pEAW1 template with primers AP307 and AP308 (SDM)
pAP104722	IgaA L472A L483A– T18 tag in pUT18	pAP10472 template with primers AP809 and AP810 (SDM)
pAP105382	IgaA L538A L483A– T18 tag in pUT18	pAP10538 template with primers AP809 and AP810 (SDM)
**RcsD plasmids:**		
pEAW7	RcsD with T18 tag at C-terminal cloned in pUT18	Wall et al., 2020[3]
pEAW7peri	RcsD Δperi$_{45-304}$ with T18 tag at C-terminal cloned in pUT18	Wall et al., 2020[3]
pEAW7s	RcsD cytoplasmic domain (326-C) with T18 tag at C-terminal cloned in pUT18	Wall et al., 2020[3]
pEAW8	RcsD with T25 tag at C-terminal cloned in pKNT25	Wall et al., 2020[3]
pEAW8peri	RcsD Δperi$_{45-304}$ with T25 tag at C-terminal cloned in pKNT25	Wall et al., 2020[3]
pEAW8s	RcsD cytoplasmic domain (326-C) with T25 tag at C-terminal cloned in pKNT25	Wall et al., 2020[3]
pEAW8T	RcsD T411A with T25 tag at C-terminal cloned in pKNT25	Wall et al., 2020[3]
pEAW11	RcsD cloned in pBAD24	Wall et al., 2020[3]
pEAW11T	RcsD T411A cloned in pBAD24	Wall et al., 2020[3]
pEAW11peri	RcsD Δperi$_{45-304}$ in pBAD24	Wall et al., 2020[3]
pAP1102	RcsD T411A Δperi (Δ45-304) cloned in pBAD24	pEAW11peri template with primers AP169 and AP170 (SDM)
pAP1107	RcsD$_{Kp}$ cloned in pBAD24 vector	pBAD24 with primers EW55fix and EW56fix; gene PCR amplified with primers AP857 and AP858
pAP7029	RcsD V29A – T18 tag in pUT18	pEAW7 template with primers AP607 and AP608 (SDM)
pAP7036	RcsD V36A – T18 tag in pUT18	pEAW7 template with primers AP867 and AP868 (SDM)
pAP7069	RcsD Y69A – T18 tag in pUT18	pEAW7 template with primers AP887 and AP888 (SDM)
pAP7134e	RcsD T134E – T18 tag in pUT18	pEAW7 template with primers AP505 and AP506 (SDM)
pAP7137	RcsD G137A – T18 tag in pUT18	pEAW7 template with primers AP873 and AP874 (SDM)
pAP7138	RcsD A138S – T18 tag in pUT18	pEAW7 template with primers AP715 and AP716 (SDM)

pAP7153	RcsD N153A – T18 tag in pUT18	pEAW7 template with primers AP831 and AP832 (SDM)
pAP7153D	RcsD N153D – T18 tag in pUT18	pEAW7 template with primers AP437 and AP438 (SDM)
pAP7191	RcsD N191A – T18 tag in pUT18	pEAW7 template with primers AP833 and AP834 (SDM)
pAP7192	RcsD A192S – T18 tag in pUT18	pEAW7 template with primers AP649 and AP650 (SDM)
pAP7193	RcsD L193A – T18 tag in pUT18	pEAW7 template with primers AP835and AP836 (SDM)
pAP7194	RcsD D194A – T18 tag in pUT18	pEAW7 template with primers AP623 and AP624 (SDM)
pAP7220	RcsD N220A – T18 tag in pUT18	pEAW7 template with primers AP219 and AP220 (SDM)
pAP7222	RcsD P222A – T18 tag in pUT18	pEAW7 template with primers AP913 and AP914 (SDM)
pAP7223	RcsD G223A – T18 tag in pUT18	pEAW7 template with primers AP915 and AP916 (SDM)
pAP7224	RcsD H224A – T18 tag in pUT18	pEAW7 template with primers AP917 and AP918 (SDM)
pAP7225	RcsD L225A – T18 tag in pUT18	pEAW7 template with primers AP309 and AP310 (SDM)
pAP7311	RcsD L311A – T18 tag in pUT18	pEAW7 template with primers AP877 and AP878 (SDM)
pAP7319	RcsD L319A – T18 tag in pUT18	pEAW7 template with primers AP613 and AP614 (SDM)
pAP7326	RcsD G326A – T18 tag in pUT18	pEAW7 template with primers AP617 and AP618 (SDM)
pAP7330	RcsD F330A – T18 tag in pUT18	pEAW7 template with primers AP885 and AP886 (SDM)
pAP7409	RcsD Q409A – T18 tag in pUT18	pEAW7 template with primers AP837 and AP838 (SDM)
pAP7410	RcsD A410G – T18 tag in pUT18	pEAW7 template with primers AP629 and AP630 (SDM)
pAP7411	RcsD T411K – T18 tag in pUT18	pEAW7 template with primers AP117 and AP118 (SDM)
pAP7412	RcsD I412A – T18 tag in pUT18	pEAW7 template with primers AP839 and AP840 (SDM)
pAP7413	RcsD N413A – T18 tag in pUT18	pEAW7 template with primers AP841and AP842 (SDM)
pAP7414	RcsD N414A – T18 tag in pUT18	pEAW7 template with primers AP843 and AP844(SDM)
pAP7415	RcsD E415A – T18 tag in pUT18	pEAW7 template with primers AP845 and AP846 (SDM)

pAP7416	RcsD L416A – T18 tag in pUT18	pEAW7 template with primers AP847 and AP848 (SDM)
pAP7919	RcsD L319A Δperi$_{45-304}$ with T18 tag in pUT18	pEAW7peri template - primers AP613 and AP614 (SDM)
pAP7929	RcsD V29A Δperi$_{45-304}$ with T18 tag in pUT18	pEAW7peri template - primers AP607 and AP608 (SDM)
pAP804	RcsD T411A Δperi$_{45-304}$ with T25 tag at C-terminal cloned in pKNT25	Petchiappan et al., 2024[4]
pAP8919	RcsD L319A Δperi$_{45-304}$ with T25 tag in pKNT25	pEAW8peri template - primers AP607 and AP608 (SDM)
pAP8929	RcsD V29A Δperi$_{45-304}$ with T25 tag in pKNT25	pEAW8peri template - primers AP613 and AP614 (SDM)
pAP11029	RcsD V29A cloned in pBAD24	pEAW11 template with primers AP607 and AP608 (SDM)
pAP11033	RcsD T33A cloned in pBAD24	pEAW11 template with primers AP865 and AP866 (SDM)
pAP11036	RcsD V36A cloned in pBAD24	pEAW11 template with primers AP867 and AP868 (SDM)
pAP11069	RcsD Y69A cloned in pBAD24	pEAW11 template with primers AP887 and AP888 (SDM)
pAP11134	RcsD T134A cloned in pBAD24	pEAW11 template with primers AP871 and AP872 (SDM)
pAP11134e	RcsD T134E cloned in pBAD24	pEAW11 template with primers AP505 and AP506 (SDM)
pAP11137	RcsD G137A cloned in pBAD24	pEAW11 template with primers AP873 and AP874 (SDM)
pAP11138	RcsD A138S cloned in pBAD24	pEAW11 template with primers AP715 and AP716 (SDM)
pAP11153	RcsD N153A cloned in pBAD24	pEAW11 template with primers AP831 and AP832 (SDM)
pAP11153D	RcsD N153D cloned in pBAD24	pEAW11 template with primers AP437 and AP438 (SDM)
pAP11191	RcsD N191A cloned in pBAD24	pEAW11 template with primers AP833 and AP834 (SDM)
pAP11192	RcsD A192S cloned in pBAD24	pEAW11 template with primers AP649 and AP650 (SDM)
pAP11193	RcsD L193A cloned in pBAD24	pEAW11 template with primers AP835and AP836 (SDM)
pAP11194	RcsD D194A cloned in pBAD24	pEAW11 template with primers AP623 and AP624 (SDM)
pAP11220	RcsD N220A cloned in pBAD24	pEAW11 template with primers AP219 and AP220 (SDM)
pAP11222	RcsD P222A cloned in pBAD24	pEAW11 template with primers AP913 and AP914 (SDM)
pAP11223	RcsD G223A cloned in pBAD24	pEAW11 template with primers AP915 and AP916 (SDM)
pAP11224	RcsD H224A cloned in pBAD24	pEAW11 template with primers AP917 and AP918 (SDM)

pAP11225	RcsD L225A cloned in pBAD24	pEAW11 template with primers AP309 and AP310 (SDM)
pAP11311	RcsD L311A cloned in pBAD24	pEAW11 template with primers AP877 and AP878 (SDM)
pAP11312	RcsD P312A cloned in pBAD24	pEAW11 template with primers AP879 and AP880 (SDM)
pAP11315	RcsD L315A cloned in pBAD24	pEAW11 template with primers AP881 and AP882 (SDM)
pAP11316	RcsD N316A cloned in pBAD24	pEAW11 template with primers AP883 and AP884(SDM)
pAP11319	RcsD L319A cloned in pBAD24	pEAW11 template with primers AP613 and AP614 (SDM)
pAP11326	RcsD G326A cloned in pBAD24	pEAW11 template with primers AP617 and AP618 (SDM)
pAP11328	RcsD T328A cloned in pBAD24	pEAW11 template with primers AP541 and AP542 (SDM)
pAP11330	RcsD F330A cloned in pBAD24	pEAW11 template with primers AP885 and AP886 (SDM)
pAP11409	RcsD Q409A cloned in pBAD24	pEAW11 template with primers AP837 and AP838 (SDM)
pAP11410	RcsD A410G cloned in pBAD24	pEAW11 template with primers AP629 and AP630 (SDM)
pAP11411	RcsD T411K cloned in pBAD24	pEAW11 template with primers AP117 and AP118 (SDM)
pAP11412	RcsD I412A cloned in pBAD24	pEAW11 template with primers AP839 and AP840 (SDM)
pAP11413	RcsD N413A cloned in pBAD24	pEAW11 template with primers AP841and AP842 (SDM)
pAP11414	RcsD N414A cloned in pBAD24	pEAW11 template with primers AP843 and AP844(SDM)
pAP11415	RcsD E415A cloned in pBAD24	pEAW11 template with primers AP845 and AP846 (SDM)
pAP11416	RcsD L416A cloned in pBAD24	pEAW11 template with primers AP847 and AP848 (SDM)
pAP113112	RcsD L311A L319A cloned in pBAD24	pAP113112 template with primers AP613 and AP614 (SDM)
pAP113192	RcsD L319A T134E cloned in pBAD24	pAP113192 template with primers AP613 and AP614 (SDM)
pAP114112	RcsD N153A T411A cloned in pBAD24	pAP114112 template with primers AP831 and AP832 (SDM)
**RcsC plasmids:**		
pEAW13	RcsC cloned in pBAD24	Wall et al., 2020[3]
pAP1312	$RcsC_{Kp}$ cloned in pBAD24 vector	pBAD24 with primers EW55fix and EW56fix; gene PCR amplified with primers AP909 and AP910
pAP13021	RcsC L21A cloned in pBAD24	pEAW13 template with primers AP893 and AP894 (SDM)
pAP13024	RcsC V24A cloned in pBAD24	pEAW13 template with primers AP921 and AP922 (SDM)

pAP13028	RcsC L28A cloned in pBAD24	pEAW13 template with primers AP895 and AP896 (SDM)
pAP13031	RcsC F31A cloned in pBAD24	pEAW13 template with primers AP897 and AP898 (SDM)
pAP13035	RcsC F35A cloned in pBAD24	pEAW13 template with primers AP899 and AP900 (SDM)
pAP13042	RcsC H42A cloned in pBAD24	pEAW13 template with primers AP901 and AP902(SDM)
pEAW26	RcsC with MalF-TM ($RcsC_{1-19}$-$MalF_{2-59}$-$RcsC_{334-C}$) in pBAD24	Wall et al., 2020[3]

Table S5: List of primers used in this study

Name	Sequence (5'-3')
AP117	CTCATACAGCTCGTTATTGATTTTCGCCTGAATAATCCCCTGAT
AP118	ATCAGGGGATTATTCAGGCGAAAATCAATAACGAGCTGTATGAG
AP137	GCGTAAACTCACACCTGAAGAACGTAGCGCC
AP138	GCCTTTCATCCATGAGAGAGTGAATTTCAGCGGCAT
AP143	GTCGGTAGAGATGCCGTACGCCGTGATAGCGTGTGTCAG
AP144	CTGACACACGCTATCACGGCGTACGGCATCTCTACCGAC
AP169	CAGCTCGTTATTGATCGCCGCCTGAATAATCCCCT
AP170	AGGGGATTATTCAGGCGGCGATCAATAACGAGCTG
AP185	GTTATCCAGCGATTTCGCACTCACCGGGC
AP186	GCCCGGTGAGTGCGAAATCGCTGGATAAC
AP213	TTCAGGCAGCTTTCAAAATAGGCCTGAATCTCCATC
AP214	GATGGAGATTCAGGCCTATTTTGAAAGCTGCCTGAA
AP219	GATGTCCTGGCTGGGCAAAGGTAGTACGCAATGTAAAGTAATG
AP220	CATTACTTTACATTGCGTACTACCTTTGCCCAGCCAGGACATC
AP299	ATTGACCAGCGCAGCTTTCAGTCGCACACAGTCATCTTTG
AP300	CAAAGATGACTGTGTGCGACTGAAAGCTGCGCTGGTCAAT
AP301	GCGATTCCGCACTCGCCGGGCGTAATAAC
AP302	GTTATTACGCCCGGCGAGTGCGGAATCGC
AP303	ATCCAGCGATTCCGCAGCCACCGGGCGTAATAAC
AP304	GTTATTACGCCCGGTGGCTGCGGAATCGCTGGAT
AP305	GGTTATCCAGCGCTTCCGCACTCACCGGG
AP306	CCCGGTGAGTGCGGAAGCGCTGGATAACC
AP307	GTTGCCACCAGGGCATCCAGCGATTCCGCACTC
AP308	GAGTGCGGAATCGCTGGATGCCCTGGTGGCAAC
AP309	CCACGACCGTTGCCGCATGTCCTGGCTGGTTAAA
AP310	TTTAACCAGCCAGGACATGCGGCAACGGTCGTGG
AP397	CCG AAT TCT TAG TTA CCC TTG TGC GTA CAT TAA AAC AAC AAG GTA ACC
AP415	CACCGGGCGTAATAACACCGCCACGCCATCGAGCTTCCC
AP416	GGGAAGCTCGATGGCGTGGCGGTGTTATTACGCCCGGTG
AP423	CATGCCGGATTTTTGAATTTCTGAACGTAATGAGGCACT

AP424	AGTGCCTCATTACGTTCAGAAATTCAAAAATCCGGCATG
AP437	ATCAGCACCAGACTGTCATCCTGACCATTCAGGTA
AP438	TACCTGAATGGTCAGGATGACAGTCTGGTGCTGAT
AP469	AATTGCTGAACGTAATGCGGCACTGACGCGAGAT
AP470	ATCTCGCGTCAGTGCCGCATTACGTTCAGCAATT
AP505	CATTTTCTGCGCCCCACAATTCATCCAGATAAGTGGACATCC
AP506	GGATGTCCACTTATCTGGATGAATTGTGGGGCGCAGAAAATG
AP541	GCGGAATGTGGCATAGCCAAATAACGCCAGCGC
AP542	GCGCTGGCGTTATTTGGCTATGCCACATTCCGC
AP553	GCCGTAACGCGTGATATCGTGTGTCAGCATCAT
AP554	ATGATGCTGACACACGATATCACGCGTTACGGC
AP559	GAG CTC GAA TTC GCT AGC CCA AAA AAA CG
AP560	AAG CTT GGC TGT TTT GGC GGA TGA G
AP561	GCCGACTAATTCTTCTTTATTGGTATAAATTCGGACCGGCGTG
AP562	CACGCCGGTCCGAATTTATACCAATAAAGAAGAATTAGTCGGC
AP589	ACCAGCGCATTTTTCAGTGCCACACAGTCATCTTTGGC
AP590	GCCAAAGATGACTGTGTGGCACTGAAAAATGCGCTGGT
AP591	GTCTTTACTGTTGCCGAGAGCGACCAGCGCATTTTTCAGT
AP592	ACTGAAAAATGCGCTGGTCGCTCTCGGCAACAGTAAAGAC
AP607	CACCCATCGTCACCAGTAACGCAATGATCAACAGTAAAAAG
AP608	CTTTTTACTGTTGATCATTGCGTTACTGGTGACGATGGGTG
AP613	CCAGCGCCAGCGCACCGATGTTCAGCAGCAGTG
AP614	CACTGCTGCTGAACATCGGTGCGCTGGCGCTGG
AP617	GCGGAATGTGGTATAGGCAAATAACGCCAGCGC
AP618	GCGCTGGCGTTATTTGCCTATACCACATTCCGC
AP623	ATTCCTGATCGTGAGCCAGCAGGCCGAGC
AP624	GCTCGGCCTGCTGGCTCACGATCAGGAAT
AP629	ACAGCTCGTTATTGATCGTACCCTGAATAATCCCCTGATG
AP630	CATCAGGGGATTATTCAGGGTACGATCAATAACGAGCTGT
AP637	GCTGAACGTAATGAGGCAGCGACGCGAGATTCATCTTC
AP638	GAAGATGAATCTCGCGTCGCTGCCTCATTACGTTCAGC
AP639	CAATACCATGCCGGCTTTTTGAATTGCTGAACGTAATGAGGC
AP640	GCCTCATTACGTTCAGCAATTCAAAAAGCCGGCATGGTATTG
AP649	GCGTTCATCGAGGGAGTTGGCCTGTTGCA
AP650	TGCAACAGGCCAACTCCCTCGATGAACGC
AP681	GTAGAGATGCCGTAACTCGTGATAGCGTGTGTC
AP682	GACACACGCTATCACGAGTTACGGCATCTCTAC
AP707	TTTGAATTGCTGAACGCGCTGAGGCACTGACGCGAGATTCATCTTC
AP708	GAAGATGAATCTCGCGTCAGTGCCTCAGCGCGTTCAGCAATTCAAA
AP709	AAATCATCAAGCAATACCATGGCGGATTTTTGAATTGCTGAAC
AP710	GTTCAGCAATTCAAAAATCCGCCATGGTATTGCTTGATGATTT
AP711	GCCAAAATCATCAAGCAATACCGCGCCGGATTTTTGAATTGCTGAA
AP712	TTCAGCAATTCAAAAATCCGGCGCGGTATTGCTTGATGATTTTGGC

AP713	GCGTTGGCGCGCTACCAGCGCGTCCCAGTCTTT
AP714	AAAGACTGGGACGCGCTGGTAGCGCGCCAACGC
AP715	CCACGGTACATTTCTGAGCCCCACAATGTATCCA
AP716	TGGATACATTGTGGGGCTCAGAAAATGTACCGTGG
AP735	CAATACCATGCCGGACGCTTGAATTGCTGAACGTAATGAGGCACTGA
AP736	TCAGTGCCTCATTACGTTCAGCAATTCAAGCGTCCGGCATGGTATTG
AP773	GTTTGCCGACTAATTCTTCTACATTGGTATAAATTCGGACC
AP774	GGTCCGAATTTATACCAATGTAGAAGAATTAGTCGGCAAAC
AP775	GAACGGTTTGCCGACATATTCTTCTGCATTGGTATAAATTCGGAC
AP776	GTCCGAATTTATACCAATGCAGAAGAATATGTCGGCAAACCGTTC
AP779	CATGCCGGATTTTTGAGCTGCTGAACGTAATGAGGCACTGA
AP780	TCAGTGCCTCATTACGTTCAGCAGCTCAAAAATCCGGCATG
AP783	CACGCCATCGAGCGCCCCGGCGTTGGCG
AP784	CGCCAACGCCGGGGCGCTCGATGGCGTG
AP785	CACATTCACGCCAGCGAGCTTCCCGGC
AP786	GCCGGGAAGCTCGCTGGCGTGAATGTG
AP787	GCGTAATAACACATTCGCGCCATCGAGCTTCCC
AP788	GGGAAGCTCGATGGCGCGAATGTGTTATTACGC
AP789	CACCGGGCGTAATAACGCATTCACGCCATCGAG
AP790	CTCGATGGCGTGAATGCGTTATTACGCCCGGTG
AP791	CACTCACCGGGCGTAATGCCACATTCACGCCATCGA
AP792	TCGATGGCGTGAATGTGGCATTACGCCCGGTGAGTG
AP809	TGTCGCCAAAATCATCAAGCGCTACCATGCCGGATTTTTGAATTG
AP810	CAATTCAAAAATCCGGCATGGTAGCGCTTGATGATTTTGGCGACA
AP813	CGTCCCAGTCTTTACTGGCGCCGAGATTGACCAGCG
AP814	CGCTGGTCAATCTCGGCGCCAGTAAAGACTGGGACG
AP815	GCACTCACCGGGGCTAATAACACATTCACGCCATCGA
AP816	TCGATGGCGTGAATGTGTTATTAGCCCCGGTGAGTGC
AP819	ACCGAGATCGCGGAACAGTTTGCCGACTAATTC
AP820	GAATTAGTCGGCAAACTGTTCCGCGATCTCGGT
AP827	AACGTAATGAGGCACTGGCGCGAGATTCATCTTCC
AP828	GGAAGATGAATCTCGCGCCAGTGCCTCATTACGTT
AP831	TGAGATCAGCACCAGACTGGCATCCTGACCATTCAGGTAAT
AP832	ATTACCTGAATGGTCAGGATGCCAGTCTGGTGCTGATCTCA
AP833	GCGTTCATCGAGGGCGGCGGCCTGTTGCAACATC
AP834	GATGTTGCAACAGGCCGCCGCCCTCGATGAACGC
AP835	CTTTCGCGTTCATCGGCGGCGTTGGCCTGTTG
AP836	CAACAGGCCAACGCCGCCGATGAACGCGAAAG
AP837	TTGATCGTCGCCGCAATAATCCCCTGATGCTGTTCCG
AP838	CGGAACAGCATCAGGGGATTATTGCGGCGACGATCAA
AP839	CTCATACAGCTCGTTATTGGCCGTCGCCTGAATAATCCCC
AP840	GGGGATTATTCAGGCGACGGCCAATAACGAGCTGTATGAG
AP841	GGATCTCATACAGCTCGTTAGCGATCGTCGCCTGAATAATCC

AP842	GGATTATTCAGGCGACGATCGCTAACGAGCTGTATGAGATCC
AP843	TCTCATACAGCTCGGCATTGATCGTCGCCTGAATAATCCC
AP844	GGGATTATTCAGGCGACGATCAATGCCGAGCTGTATGAGA
AP845	GCGGATCTCATACAGCGCGTTATTGATCGTCGC
AP846	GCGACGATCAATAACGCGCTGTATGAGATCCGC
AP847	GAACATGCGGATCTCATACGCCTCGTTATTGATCGTCGCC
AP848	GGCGACGATCAATAACGAGGCGTATGAGATCCGCATGTTC-
AP857	AGC AGG AGG AAT TCA ATG ACC CCG AAA AAA TTC TCC CTG ATC C
AP858	AAA ACA GCC AAG CTT CTA CAG CAA GCT CTT GAC GTA AGC GTC
AP865	GTACCATTACACCCATCGCCACCAGTAACACAATGAT
AP866	ATCATTGTGTTACTGGTGGCGATGGGTGTAATGGTAC
AP867	GGCGCTTTGTACCATTGCACCCATCGTCACCAG
AP868	CTGGTGACGATGGGTGCAATGGTACAAAGCGCC
AP871	TCTGCGCCCCACAATGCATCCAGATAAGTGGAC
AP872	GTCCACTTATCTGGATGCATTGTGGGGCGCAGA
AP873	CGGTACATTTTCTGCGGCCCACAATGTATCCAG
AP874	CTGGATACATTGTGGGCCGCAGAAAATGTACCG
AP877	GATGTTCAGCAGCAGTGGCGCCAGAATGTTTTGCAACGTA
AP878	TACGTTGCAAAACATTCTGGCGCCACTGCTGCTGAACATC
AP879	GATGTTCAGCAGCAGTGCCAGCAGAATGTTTTGCA
AP880	TGCAAAACATTCTGCTGGCACTGCTGCTGAACATC
AP881	CAGCAAACCGATGTTCGCCAGCAGTGGCAGCAGA
AP882	TCTGCTGCCACTGCTGGCGAACATCGGTTTGCTG
AP883	GCCAGCAAACCGATGGCCAGCAGCAGTGGCAG
AP884	CTGCCACTGCTGCTGGCCATCGGTTTGCTGGC
AP885	GGAGAAATGGCGGGCTGTGGTATAGCCAAATAACGCCAGC
AP886	GCTGGCGTTATTTGGCTATACCACAGCCCGCCATTTCTCC
AP887	CTGCCAGGTCACGGCACGCCAGTTATCGACGCG
AP888	CGCGTCGATAACTGGCGTGCCGTGACCTGGCAG
AP891	TAATAACACATTCACGGCATCGAGCTTCCCGGC
AP892	GCCGGGAAGCTCGATGCCGTGAATGTGTTATTA
AP893	AGAGCACTAACGCCGCTGCTCTGAACATGTAGCGCG
AP894	CGCGCTACATGTTCAGAGCAGCGGCGTTAGTGCTCT
AP895	AACGGATGAAAAAGCAATCGCCAGCCAGAGCACTAACGCC
AP896	GGCGTTAGTGCTCTGGCTGGCGATTGCTTTTTCATCCGTT
AP897	ACGATGTAAAAAACGGATGAAGCAGCAATCAACAGCCAGAGCAC
AP898	GTGCTCTGGCTGTTGATTGCTGCTTCATCCGTTTTTTACATCGT
AP899	CTGATGTAACGCATTAACGATGTAAGCAACGGATGAAAAAGCAATCAACAGC
AP900	GCTGTTGATTGCTTTTTCATCCGTTGCTTACATCGTTAATGCGTTACATCAG
AP901	CCGATTCTCGCTGAGCTAACGCATTAACGATGTAAAAAACGGA
AP902	TCCGTTTTTTACATCGTTAATGCGTTAGCTCAGCGAGAATCGG
AP903	CGAGATCGCGGAACGGTGCGCCGACTAATTCTTCTG
AP904	CAGAAGAATTAGTCGGCGCACCGTTCCGCGATCTCG

AP905	CTTCACCGAGATCGCGGGCCGGTTTGCCGACTAATT
AP906	AATTAGTCGGCAAACCGGCCCGCGATCTCGGTGAAG
AP907	ACTTCACCGAGATCGGCGAACGGTTTGCCGAC
AP908	GTCGGCAAACCGTTCGCCGATCTCGGTGAAGT
AP909	AGC AGG AGG AAT TCA ATG AAA TAT CTT GCC TCT TTT CAT ACA ACC CTG AAG
AP910	AAA ACA GCC AAG CTT TTA TTC CCG CCC TTT ACG CAC CCG
AP913	CGTTGCCAGATGTCCTGCCTGGTTAAAGGTAGTAC
AP914	GTACTACCTTTAACCAGGCAGGACATCTGGCAACG
AP915	ACCGTTGCCAGATGTGCTGGCTGGTTAAAGG
AP916	CCTTTAACCAGCCAGCACATCTGGCAACGGT
AP917	ACGACCGTTGCCAGAGCTCCTGGCTGGTTAAAGG
AP918	CCTTTAACCAGCCAGGAGCTCTGGCAACGGTCGT
AP923	AAAAGCAATCAACAGCCAGGCCACTAACGCCAATGCTCTG
AP924	CAGAGCATTGGCGTTAGTGGCCTGGCTGTTGATTGCTTTT
AP939	TTTACTGTTGCCGGCATTGACCAGCGCATTTTTCAGTCGC
AP940	GCGACTGAAAAATGCGCTGGTCAATGCCGGCAACAGTAAA
EW3	TAC CCG GGG ATC CTC TAG AGT CG
EW4	TAA CTA AGA ATT CGG CCG TCG TTT TAC AAC GTC
EW55fix	TGA ATT CCT CCT GCT AGC CCA AAA AAA CGG GTA TGG AG
EW56fix	AAG CTT GGC TGT TTT GGC GGA TGA GAG AAG ATT TTC AGC
EW81	GCT ATG ACC ATG ATT ACC CCG AAA AAA TTC TCC CTG ATC CCC
EW82	GCA GGC ATG CAA GCT CAG CAA GCT CTT GAC GTA AGC GTC
EW213	ACCACGCCTGACAGACTAAGTAAGATGGGGAAAGC ATG AGC ACC ATT GTG ATT TTT TTA GCT GCT TTG C
EW214	GACAGGGTAGCATAACCTGCCGCGCAAACGTG TTA TTC GAT AAG GCT TTC TGA AGG GGT GAT CAG TTG

Table S6: List of synthetic gene fragments (gBlocks) used in this study

**Name**	**Sequence (5'-3')**
AP_FMF25kp	GAGGATCCCCGGGTAATGGATGTCATTAAAAAGAAACATTGGTGG CAAAGCGACGCGCTGAAATGGTCAGTGCTAGGTCTGCTCGGCCTG CTGGTGGGTTACCTTGTTGTTTAATGTACGCACAAGGGTCCATGC TAAGCAGATCTCCCGTTGAGCCTGCTCAAAGCACTGCAACCCCGCC GGTTAAATCTGAGCCGAGCAAACCGCGCGCCACCCGCCCGGCCCC AGTACGTATTTACACCGATGCGTCAGAGCTGGTTGGTAAACCGTTC CGCGATCTGGGTGAAGTATCTGGCGAGTCCTGCCAGGCTTCGAATC AGGATTCTCCGCCGAATATCCCCACCGCGCGCAAGCGCCTGCAAA TTAATGCCGCGCGTATGAAAGCTAATGCCGTCCTGCTTCACCGCTG CGAAGTGACCAGCGGTACGCCAGGCTGCTACCGTCAGGCCGTTTG CCTGGGTTCAGCGCTTAACGTCTCGGCGCAATAACTAAGAATTCGG

AP_GF33kp	AGCGAATTCGAGCTCAGCAGGAGGAATTCAATGCGTGCTTTACCG ATCTGTTTGTTAGCACTCATGTTAAGCGGTTGTTCCATGCTAAGCA GATCTCCCGTTGAGCCTGCTCAAAGCACTGCAACCCCGCCGGTTAA ATCTGAGCCGAGCAAACCGCGCGCCACCCGCCCGGCCCCAGTACG TATTTACACCGATGCGTCAGAGCTGGTTGGTAAACCGTTCCGCGAT CTGGGTGAAGTATCTGGCGAGTCCTGCCAGGCTTCGAATCAGGATT CTCCGCCGAATATCCCCACCGCGCGCAAGCGCCTGCAAATTAATG CCGCGCGTATGAAAGCTAATGCCGTCCTGCTTCACCGCTGCGAAGT GACCAGCGGTACGCCAGGCTGCTACCGTCAGGCCGTTTGCCTGGG TTCAGCGCTTAACGTCTCGGCGCAATGAAAGCTTGGCTGTTTT

Table S7: Software and algorithms

Name	Description	Reference
CryoSPARC	Cryo-EM data processing software	Punjani et. al 2017[9]
TOPAZ	Cryo-EM data particle picking software	Bepler et. al 2019[10]
PHENIX	Structure refinement and analysis software	Liebschner et. al 2019[11]
BioWulf Cluster	Data processing and storage	NIH HPC[12]
DeepEMhancer	Cryo-EM map improvement software	Sanchez-Garcia et. al 2021[13]
ChimeraX	Structure analysis and image preparation software	Meng et. al 2023[14]
Chimera	Structure analysis and image preparation software	Pettersen et. al 2004[15]
GraphPad Prism	Data processing software	GraphPad Prism[16]
EPU	Data collection software	ThermoFisher
SerialEM	Data collection software	Mastronarde et. al[17]
Coot	Model building software	Emsley et. al 2010[18]
AlphaFold 3	Structure prediction	Abramson et. al 2024[19]
Isolde	Model building software	Croll[20]

Supplemental References

1. Madeira, F., Madhusoodanan, N., Lee, J., Eusebi, A., Niewielska, A., Tivey, A.R.N., Meacham, S., Lopez, R., and Butcher, S. (2024). Using EMBL-EBI Services via Web Interface and Programmatically via Web Services. Curr Protoc *4*, e1065. 10.1002/cpz1.1065.
2. Karimova, G., Pidoux, J., Ullmann, A., and Ladant, D. (1998). A bacterial two-hybrid system based on a reconstituted signal transduction pathway. Proc Natl Acad Sci U S A *95*, 5752-5756. 10.1073/pnas.95.10.5752.
3. Wall, E.A., Majdalani, N., and Gottesman, S. (2020). IgaA negatively regulates the Rcs Phosphorelay via contact with the RcsD Phosphotransfer Protein. PLoS Genet *16*, e1008610. 10.1371/journal.pgen.1008610.
4. Petchiappan, A., Majdalani, N., Wall, E., and Gottesman, S. (2024). RcsF-independent mechanisms of signaling within the Rcs phosphorelay. PLoS Genet *20*, e1011408. 10.1371/journal.pgen.1011408.
5. Guzman, L.M., Belin, D., Carson, M.J., and Beckwith, J. (1995). Tight regulation, modulation, and high-level expression by vectors containing the arabinose PBAD promoter. J Bacteriol *177*, 4121-4130. 10.1128/jb.177.14.4121-4130.1995.
6. Cherepanov, P.P., and Wackernagel, W. (1995). Gene disruption in Escherichia coli: TcR and KmR cassettes with the option of Flp-catalyzed excision of the antibiotic-resistance determinant. Gene *158*, 9-14. 10.1016/0378-1119(95)00193-a.
7. Karimova, G., Ullmann, A., and Ladant, D. (2001). Protein-protein interaction between Bacillus stearothermophilus tyrosyl-tRNA synthetase subdomains revealed by a bacterial two-hybrid system. J Mol Microbiol Biotechnol *3*, 73-82.
8. Karimova, G., Dautin, N., and Ladant, D. (2005). Interaction network among Escherichia coli membrane proteins involved in cell division as revealed by bacterial two-hybrid analysis. J Bacteriol *187*, 2233-2243. 10.1128/JB.187.7.2233-2243.2005.
9. Punjani, A., Rubinstein, J.L., Fleet, D.J., and Brubaker, M.A. (2017). cryoSPARC: algorithms for rapid unsupervised cryo-EM structure determination. Nat Methods *14*, 290-296. 10.1038/nmeth.4169.
10. Bepler, T., Morin, A., Rapp, M., Brasch, J., Shapiro, L., Noble, A.J., and Berger, B. (2019). Positive-unlabeled convolutional neural networks for particle picking in cryo-electron micrographs. Nat Methods *16*, 1153-1160. 10.1038/s41592-019-0575-8.
11. Liebschner, D., Afonine, P.V., Baker, M.L., Bunkoczi, G., Chen, V.B., Croll, T.I., Hintze, B., Hung, L.W., Jain, S., McCoy, A.J., et al. (2019). Macromolecular structure determination using X-rays, neutrons and electrons: recent developments in Phenix. Acta Crystallogr D Struct Biol *75*, 861-877. 10.1107/S2059798319011471.
12. This work utilized the computational resources of the NIH HPC Biowulf cluster (https://hpc.nih.gov), B.M.U.
13. Sanchez-Garcia, R., Gomez-Blanco, J., Cuervo, A., Carazo, J.M., Sorzano, C.O.S., and Vargas, J. (2021). DeepEMhancer: a deep learning solution for cryo-EM volume post-processing. Commun Biol *4*, 874. 10.1038/s42003-021-02399-1.
14. Meng, E.C., Goddard, T.D., Pettersen, E.F., Couch, G.S., Pearson, Z.J., Morris, J.H., and Ferrin, T.E. (2023). UCSF ChimeraX: Tools for structure building and analysis. Protein Sci *32*, e4792. 10.1002/pro.4792.
15. Pettersen, E.F., Goddard, T.D., Huang, C.C., Couch, G.S., Greenblatt, D.M., Meng, E.C., and Ferrin, T.E. (2004). UCSF Chimera--a visualization system for exploratory research and analysis. J Comput Chem *25*, 1605-1612. 10.1002/jcc.20084.
16. GraphPad Prism version 10.0.0 GraphPad Software, Boston, Massachusetts USA, www.graphpad.com.
17. Mastronarde, D.N. (2005). Automated electron microscope tomography using robust prediction of specimen movements. J Struct Biol *152*, 36-51. 10.1016/j.jsb.2005.07.007.

18. Emsley, P., Lohkamp, B., Scott, W.G., and Cowtan, K. (2010). Features and development of Coot. Acta Crystallogr D Biol Crystallogr *66*, 486-501. 10.1107/S0907444910007493.
19. Abramson, J., Adler, J., Dunger, J., Evans, R., Green, T., Pritzel, A., Ronneberger, O., Willmore, L., Ballard, A.J., Bambrick, J., et al. (2024). Accurate structure prediction of biomolecular interactions with AlphaFold 3. Nature *630*, 493-500. 10.1038/s41586-024-07487-w.
20. Croll, T.I. (2018). ISOLDE: a physically realistic environment for model building into low-resolution electron-density maps. Acta Crystallogr D Struct Biol *74*, 519-530. 10.1107/S2059798318002425.
